# PTEN expression by an oncolytic herpesvirus directs T-cell mediated tumor clearance

**DOI:** 10.1038/s41467-018-07344-1

**Published:** 2018-11-27

**Authors:** Luke Russell, Jessica Swanner, Alena Cristina Jaime-Ramirez, Yufeng Wang, Alex Sprague, Yeshavanth Banasavadi-Siddegowda, Ji Young Yoo, Gina M. Sizemore, Raleigh Kladney, Jianying Zhang, Norman L. Lehman, Michael C Ostrowski, Bangxing Hong, Michael Caligiuri, Jianhua Yu, Balveen Kaur

**Affiliations:** 1 Department of Neurological Surgery, Ohio State University Comprehensive Cancer Center, Columbus, 43210 OH USA; 2 Department of Hematology & Hematopoietic Cell Transplantation,, City of Hope National Medical Center and Beckman Research Institute, Duarte, 91010 CA USA; 3 Department of Neurosurgery, McGovern Medical School, University of Texas Health Science Center at Houston, Houston, 77030 TX USA; 4 Surgical Neurology Branch, NINDS, National Institutes of Health, Bethesda, 20852 MD USA; 5 Department of Radiation Oncology, Ohio State University Comprehensive Cancer Center, Columbus, 43210 OH USA; 6 Department of Molecular Genetics, Ohio State University Comprehensive Cancer Center, 43210, Columbus, OH USA; 7 Division of Biostatistics, Department of Information Sciences, City of Hope National Medical Center, Duarte, 91010 California USA; 8 Department of Pathology and Laboratory Medicine, The Brown Cancer Center, University of Louisville, Louisville, 40202 KY USA; 9 Hollings Cancer Center, Department of Biochemistry and Molecular Biology, Medical University of South Carolina, Charleston, 29425 SC USA

## Abstract

Engineered oncolytic viruses are used clinically to destroy cancer cells and have the ability to boost anticancer immunity. Phosphatase and tensin homolog deleted on chromosome 10 loss is common across a broad range of malignancies, and is implicated in immune escape. The N-terminally extended isoform, phosphatase and tensin homolog deleted on chromosome 10 alpha (PTENα), regulates cellular functions including protein kinase B signaling and mitochondrial adenosine triphosphate production. Here we constructed HSV-P10, a replicating, PTENα expressing oncolytic herpesvirus, and demonstrate that it inhibits PI3K/AKT signaling, increases cellular adenosine triphosphate secretion, and reduces programmed death-ligand 1 expression in infected tumor cells, thus priming an adaptive immune response and overcoming tumor immune escape. A single dose of HSV-P10 resulted in long term survivors in mice bearing intracranial tumors, priming anticancer T-cell immunity leading to tumor rejection. This implicates HSV-P10 as an oncolytic and immune stimulating therapeutic for anticancer therapy.

## Abstract (web summary)

Oncolytic viruses are a promising therapeutic approach for cancer treatment. The authors demonstrate the efficacy of an engineered HSV-1 expressing PTENα as an oncolytic and immune stimulating therapy against brain cancer metastases.

## Introduction

While malignant brain tumors affect over 138,000 patients in the USA, treatment options for these patients remain sparse and prognoses are consistently poor^1–3^. Both primary and metastatic malignant brain tumors are treated with total surgical resection of the bulk tumor mass followed by a combination of chemotherapy and radiation therapy^4–6^. Brain metastatic tumors account for the majority of malignant brain tumors, and for patients with breast cancer brain metastases, the second most common brain metastasizing tumor type and primary cause of malignant brain tumors in women, standard of care provides median survival rates ranging from 2 to 21 months^1,6–8^. The poor overall survival of brain tumor patients diagnosed with primary or metastatic cancer indicates a strong need for novel therapeutic discoveries and innovative therapies.

Oncolytic viral therapy is one such innovative therapy that is finding increasing use in the clinic as a therapy with multimodal benefits: direct tumor cell lysis and a method to boost anticancer immunity through the pathogen response to viral infection^9,10^. Oncolytic viruses are often engineered to remove virulent genes, and maintain replication competency in cancer cells leading to tumor specific lytic destruction^9^. Second generation oncolytic viruses have been armed with therapeutic transgenes inserted into the viral genome to boost oncolytic efficacy and provide an additional benefit to the patient. Therapeutic transgene selection can be used to increase viral spread^11–15^, enhance tumor cell killing^16,17^, trigger the death of prodrug containing tumor cells^13^, or recruit immune cells to boost antitumor immunity^18,19^. To date, many oncolytic viruses have been clinically tested, spanning multiple viral backbones, with engineered oncolytic Herpes Simplex Virus type 1 (HSV1) being the first and only virus to date to gain Food and Drug Administration (FDA) approval^20^. Numerous preclinical approaches have been reported to improve efficacy when combining oncolytic viruses with immune-boosting checkpoint inhibitor therapies^19,21–26^, which has led to the initiation of several clinical trials evaluating the safety and efficacy of this approach in patients (NCT03069378, NCT02626000, NCT02263508, NCT02798406, NCT02879760, NCT03003676, NCT03004183, NCT03153085, NCT02977156, NCT03003676^10,27,28^,).

Phosphatase and tensin homolog deleted on chromosome 10 (*PTEN*) encodes for a lipid/protein phosphatase and is characterized as a tumor suppressor gene for its role in antagonizing the Phosphatidyl inositol-4,5-bisphosphate 3-kinase/Protein kinase B (PI3K/AKT) pathway^29^. *PTEN* loss is prevalent across a variety of tumors including those originating from the bladder, prostate, brain, breast, and ovary.^29–32^. *PTEN* loss is also frequently observed in breast cancer brain metastases and is frequently lost in both human and mouse tumors that metastasize to the brain. Cancer cells that lose PTEN protein expression demonstrate increased AKT pathway activity and show increased cellular survival, proliferation, and protein synthesis, as well as increased resistance to T-cell based therapies making the PI3K/AKT pathway a frequent target of anticancer drugs^32–35^. PI3K antagonists have traditionally had limited success clinically;^33,36,37^ however, in 2014 the FDA granted approval of the PI3K inhibitor Idelalisib for the treatment of relapsed chronic lymphocytic leukemia, follicular lymphoma, and small lymphocytic lymphoma^38^, indicating that there is clinical promise for PI3K inhibitor therapy. A recently discovered N-terminally extended isoform of PTEN, PTENα, has been shown to play multiple roles inside the cell: it performs the phospholipid phosphatase function of canonical PTEN^39^, as well as localizing to cytochrome C in mitochondria where it acts to drive electron transport chain activity, resulting in increased adenosine triphosphate (ATP) production^40^.

Given the frequent loss of PTEN observed in brain disseminating tumors, we hypothesized that expression of PTEN into these tumors via oncolytic virus would improve anticancer efficacy. To our knowledge, the impact of PTENα expression during lytic viral replication in cancer cells has not been investigated. Our findings disclose that a PTENα expressing virus efficiently lyses the bulk tumor mass while creating an ATP-rich immune stimulating microenvironment during infection, and also decreases cell surface PD-L1 expression on the surface of tumor cells after treatment. We conclude that reconstitution of PTENα expression during oncolysis enhances the development of antitumor immunity in a multi-mechanistic manner, and hence improves response to virotherapy.

## Results

### Construction of PTENα expressing virus: HSV-P10

**Fig. 1:**
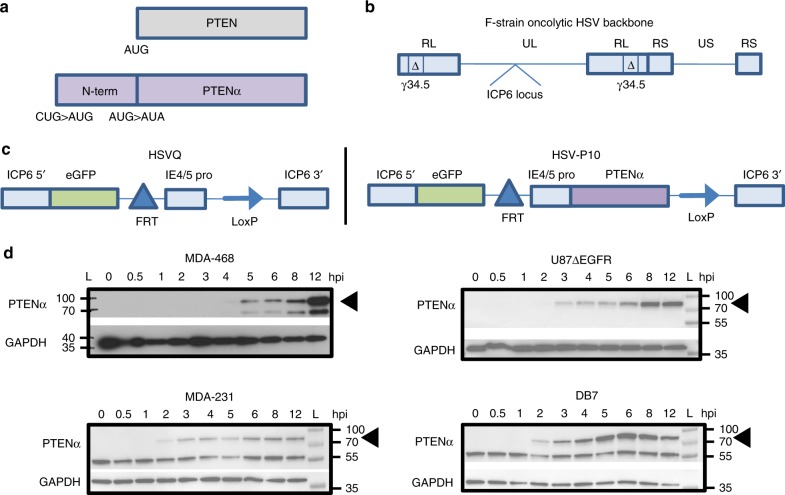
HSV-P10 construction and characterization. **a** Graphical representation of PTEN and PTENα coding sequences showing the mutations engineered in PTENα start codons to enhance translation of full-length PTENα. **b**–**c** Graphical representation of the DNA structure of wild-type F-strain HSV1 backbone showing doubly deleted γ34.5 genes, and gene-disrupting insertional ICP6 locus containing an enhanced green fluorescent protein (eGFP) gene with PTENα insertion (HSV-P10) or without PTENα (HSVQ). **d** PTENα production in infected tumor cell lysates over time. The indicated tumor cells were infected with HSV-P10 at MOI = 0.5 and harvested 0–12 hpi and probed for PTEN by western blot

We constructed a *PTENα* expressing oncolytic virus, HSV-P10, using a modified *PTENα* gene sequence, whereby we mutated the *PTENα* CUG start codon to AUG to enhance translation of the full-length N-terminally extended protein, and the internal canonical *PTEN* AUG start codon to AUA to abrogate canonical PTEN expression from the construct (Fig. 1a)^39^. We incorporated *PTENα* into an oncolytic HSV1 backbone deleted for both copies of γ34.5 within the ICP6 gene locus of the virus. Fig. 1b shows the structure of the genetic backbone of oncolytic F-strain HSV1, and Fig. 1c depicts the structure of the genetic manipulations engineered within the ICP6 locus in the control (HSVQ) and HSV-P10 viruses used in this study. To validate HSV-P10 expression of PTENα, breast cancer and glioma cells infected with HSV-P10 were probed for expression of the PTENα protein at the indicated time points post infection (Fig. 1d).

### Functionality of PTENα expressed by HSV-P10

**Fig. 2:**
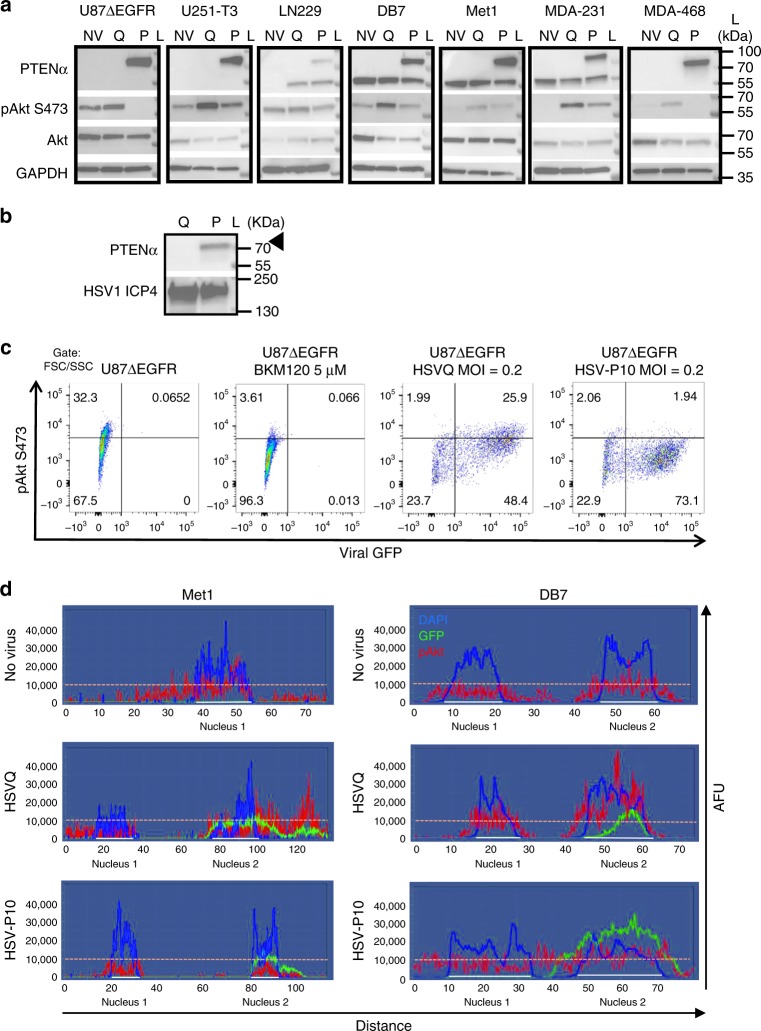
HSV-P10 AKT inhibition. **a** Western blots of lysates from the indicated cells infected with HSVQ (Q) or HSV-P10 (P) or left untreated (NV). Infected cells (MOI = 1) were lysed 24 hpi and probed for expression of PTENα, pAKT (S473), Akt, and GAPDH. L indicates the moleculcar weight ladder. **b** Western blot for secreted PTEN expression in conditioned medium. Culture media was concentrated 20X from U87∆EGFR cells infected 24 h with either HSVQ (Q) or HSV-P10 (P10) and probed for secreted PTENα. L indicates the molecular weight ladder. **c** Flow cytometric analysis of pAkt-S473 and virus encoded GFP in infected U87∆EGFR cells 15 hpi. **d** Spectral analysis of fluorescent intensity along a random line drawn across the view field of one or two cells visualizing pAKT (red), DAPI (blue) and GFP (green) of the indicated cells. *y*-axis represents arbitrary fluorescence units (AFU). Note the higher intensity of pAKT in HSVQ-infected (GFP positive) cells relative to adjacent uninfected (GFP negative) cell (middle panel). Note the lack of increased pAKT intensity in HSV-P10 infected (GFP positive) cells relative to adjacent uninfected (GFP negative) cells (bottom panel)

**Fig. 3:**
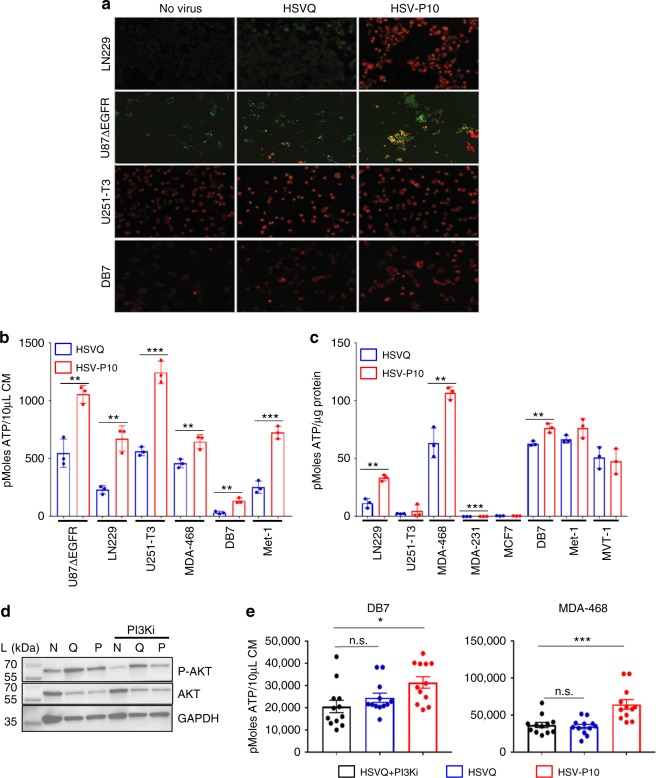
Effects of PTENα on HSV-P10 infected cells. **a** Increased mitochondrial membrane potential in HSV-P10 infected cells 6 hpi. Seeded cells were infected with either HSVQ or HSV-P10 at MOI = 0.5 for 6 h, and mitochondrial membrane potential indicated by JC-1 red fluorescence was imaged using fluorescence microscopy. Scale bar = 100 µm. **b** ATP release from infected cells 24 hpi. Data shown are mean ATP concentration in conditioned media from infected cells 24 hpi ± S.D. Statistical analysis performed with a 2-tailed Student’s *T*-test. (*n* = 3/group, **p < 0.01, ***p < 0.001). **c** ATP measured in cell lysates of infected cells 24 hpi. Data shown are mean ATP concentration 24 hpi ± S.D. Statistical analysis performed with a 2-tailed Student’s *T*-test. (*n* = 3/group, **p < 0.01, ***p < 0.001). **d** Western blot of DB7 cells 24 hpi with HSVQ (Q), HSV-P10 (P), or left untreated (NV) ± PI3Ki (LY294002). L indicates the molecular weight ladder. **e** ATP release from DB7 or MDA-468 cells 12 hpi with HSVQ or HSV-P10. HSVQ and PI3Ki treated samples were also evaluated for ATP release. Data shown are mean ATP concentrations in conditioned media from infected cells ± SEM. Statistical analysis performed with a 2-tailed Student’s *T*-test (*n* = 6/group, *p < 0.05, ***p < 0.001, n.s. = not significant)

To evaluate the functionality of PTENα expressed by HSV-P10, we tested the impact of its infection on the PI3K/AKT signaling pathway^39^. Western blot analysis of infected cells revealed increased phosphorylation of AKT upon infection with control HSVQ virus in all breast and glioma tumor cell lines tested, while HSV-P10 infected cells revealed reduced phosphorylated AKT compared with control virus infection (Fig. 2a). Apart from functioning as an intracellular lipid phosphatase that can regulate PI3K signaling, PTENα has been shown to be secreted and then re-enter adjacent cells to inhibit PI3K signaling^39^ and also to activate the mitochondrial electron transport chain through hyper-activation of cytochrome C, resulting in increased mitochondrial membrane potential and ATP production^40^. HSV-P10 infection did result in a small amount of secreted PTENα that could be detected in concentrated (20×) conditioned medium of infected U87∆EGFR cells (Fig. 2b). Additionally, immunofluorescent staining of pAKT in Met1 and DB7 cells infected with HSVQ or HSV-P10 revealed that while HSVQ infection increased pAKT staining within GFP positive cells, HSV-P10 infection did not (Supplementary Figure 1). Spectral quantification of immunofluorescent images across a straight line encompassing two cell widths of two representative adjacent cells, confirmed an increase in pAKT (red) staining within GFP positive (green) HSVQ-infected cells compared with neighboring GFP negative cells (Fig. 2d middle panel). HSV-P10 infection did not reveal infection associated induction of pAKT (Fig. 2d bottom panel). Similar results were observed in the DB7 murine breast cancer cells (Fig. 2d). Next we tested the impact of HSV-P10 on mitochondrial membrane potential using membrane-permeant JC-1 dye. This dye shows a potential-dependent mitochondrial accumulation resulting in aggregates that display a fluorescence emission shift from green (~529 nm) to red (~590 nm). Consistent with the known function of PTENα, we observed an increase in mitochondrial membrane potential in three (LN229, U87ΔEGFR, and DB7) out of four cell lines infected with HSV-P10 relative to control HSVQ-infected cells (Fig. 3a). U251T3 glioma cells possessed a high mitochondrial membrane potential prior to infection, and did not show a further change after HSV-P10 infection. The HSV-P10 induced change in mitochondrial membrane potential positively affected overall ATP production observed in both secreted conditioned medium (Fig.3b) and cell lysates (Fig. 3c). To evaluate the significance of HSV-P10 mediated inhibition of AKT phosphorylation on increased ATP production we repeated the above experiment in cells treated with and without LY294002 a PI-3 Kinase inhibitor. Western blot analysis confirmed the reduction of pAKT in LY294002 treated infected and uninfected cells (Fig. 3d). While HSV-P10 treatment increased ATP production over HSVQ, treatment with LY294002 did not increase ATP release (Fig. 3e). Collectively these results show that HSV-P10 expressed PTENα, retained both mitochondrial targeting and PI3K inhibitory functions of PTEN, and that the ATP release mediated by HSV-P10 was independent of its effect on AKT phosphorylation.

### PTENα does not inhibit HSV-P10 kinetics

**Fig. 4:**
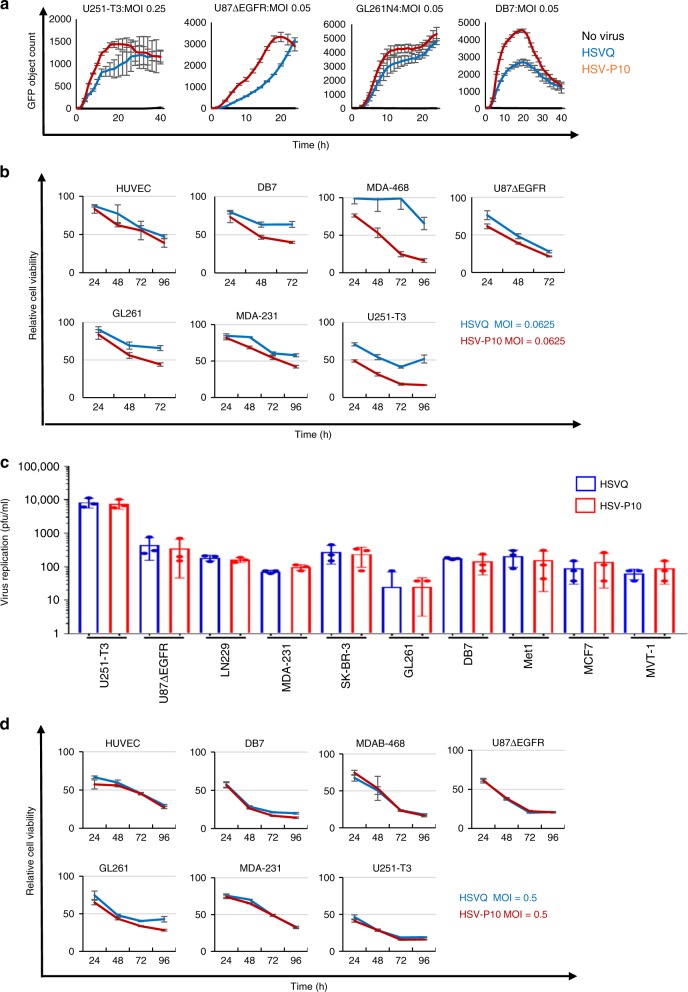
Kinetics of HSV-P10. **a** Comparison of HSVQ and HSV-P10 virus replication in the indicated cells. Seeded tumor cells were infected at the designated MOIs and GFP expression was monitored for 24–48 h utilizing the Cytation 5 Cell Imaging Multi-Mode Reader in conjunction with a BioSpa 8 Automated Incubator (BioTek Instruments, INC.). GFP object count was quantified and graphed as an average of 4 wells per treatment group ± SEM. Black line: no virus, blue line: HSVQ, red line: HSV-P10. **b** Cytolytic activity of HSVQ (blue line) vs. HSV-P10 (red line) at a low MOI. Cell viability of HSVQ or HSV-P10 infected tumor cell lines at MOI = 0.0625 for all cells at the indicated time points after infection as measured by MTT. Data shown are percentage of viable cells relative to uninfected controls ± S.D. (*n* = 6). **c** Comparison of HSVQ and HSV-P10 viral yield (burst size). Data shown are median titers from cultures ± S.D. (*n* = 3/group). **d** Cytolytic activity of HSVQ (blue line) vs. HSV-P10 (red line) at high MOI. Cell viability of HSVQ or HSV-P10 infected tumor cell lines at MOI = 0.5 for all cells at the indicated time points after infection as measured by MTT. Data shown are percentage of viable cells relative to uninfected controls ± S.D. (*n* = 6)

To test whether the expression of PTENα affected viral replication and/or tumor cell killing, we infected the indicated tumor cells with either control HSVQ (blue) or HSV-P10 (red) and monitored virus infection by quantifying GFP expression in infected cells by real time live cell fluorescent imaging using a Cytation 5 Cell Imaging Multi-Mode Reader in conjunction with a BioSpa 8 Automated Incubator (BioTek Instruments, INC.) over time (Fig. 4a). While HSV-P10 appeared to have superior kinetics of virus replication (Fig. 4a), it did not appear to correlate with PTEN and or activated base line AKT status of these cells in this small sample size (Supplementary 1). The faster kinetics implied that cells infected with HSV-P10 would burst faster than control HSVQ-infected cells. Consistent with this, tumor cells were more sensitive to HSV-P10 induced killing at lower MOIs than control HSVQ-infected cells (Fig. 4b). Next we measured virus yield from cultures infected with either HSV-P10 or control HSVQ. While HSV-P10 did appear to improve the kinetics of virus replication in infected cells (Fig. 4a), it did not appear to significantly affect the burst size or total virus yield in (Fig. 4c). Consistent with a similar burst size of the viruses, the killing of tumor cells at higher MOIs, when a significant portion of the culture is infected, was found to be similar for both the viruses (Fig. 4d).

**Fig. 5:**
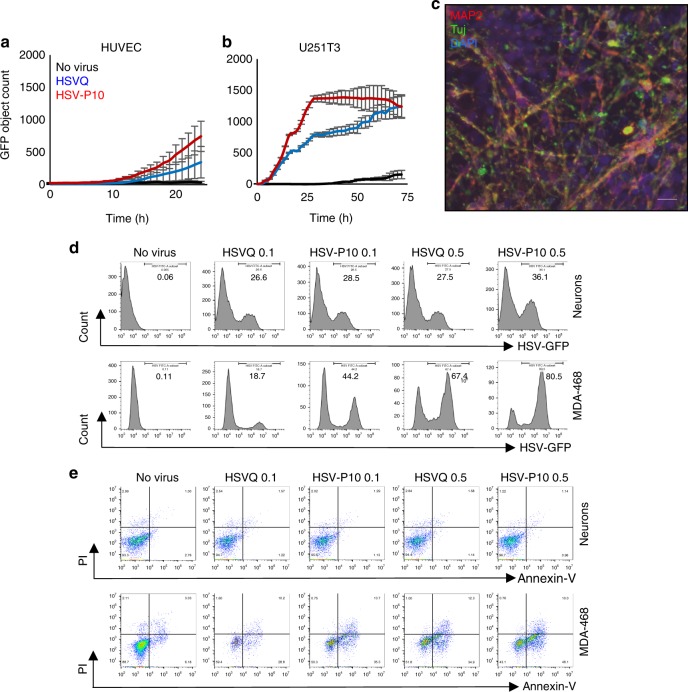
Safety of HSV-P10. Viral spread of HSVQ (blue line) and HSV-P10 (red line) in cultures of HUVEC (**a**) and U251T3 tumor cells (**b**) was assessed using the Cytation 5 live imaging system, where GFP expression was monitored over time. GFP object count was quantified and graphed as an average of 4 wells per treatment group ± SEM. **c** Immunofluorescent staining of differentiated neurons showing MAP2 and Tuj1 staining. Scale bar = 50 µm. **d** Infection (GFP) of neurons and MDA468 cells followed by GFP visualization. Data shown are histograms of the indicated cells infected with HSVQ or HSV-P10. **e** Cytolytic activity (PI/Annexin-V) in neurons was assessed by flow cytometry 24 hpi. Dot blots of annexin and PI staining of infected (GFP positive) neurons and MDA-468 cells

To evaluate the safety of an oHSV encoding for PTENα, we compared the infection and toxicity of HSVQ and HSV-P10 towards normal HUVEC and human neuronal cells. Briefly the spread of viral infection in normal HUVEC and U251T3 glioma cells was monitored over time relative to cancer cells by quantifying GFP expression in infected cells by real time live cell fluorescent imaging. While both HSVQ and HSV-P10 showed evidence of rapid virus spread through the culture in U251 cancer cells, this was highly attenuated in primary normal HUVEC cell cultures (Fig. 5a, b). Primary neuronal cultures differentiated from iPSCs derived neuron stem cells were cultured in matrigel-coated plates in neurobasal media containing BDNF (10 ng/ml, Peprotech) and GDNF (10 ng/ml, Peprotech) for 4 weeks. The differentiation of cells to neurons was analyzed by immunofluorescence staining for microtubule-associated protein 2 (MAP2) and neuron-specific beta-III tubulin (Tuj-1) (Fig. 5c). The differentiated neurons and MDA-468 cells were infected with HSVQ or HSV-P10 with MOI 0.1 or MOI 0.5 for 72 h. As expected both HSVQ and HSVP-10 could infect primary human neurons, and consistent with increased HSV-P10 spread in cancer cell cultures, a greater number of MDA-MB-468 cells were positive for GFP at either MOI, with HSV-P10 relative to HSVQ (Fig. 5d). The apoptosis of infected neurons and MDA-468 cells was analyzed by Annexin-V/PI staining of HSV-infected neurons (Gating on MAP2 + HSV-GFP + cells, Supplementary Figure 2). Consistent with the known clinical safety of HSV viruses deleted for viral neurovirullence genes ICP34.5, we observed no evidence of increased apoptosis towards these cells in vitro (Fig. 5e). This suggests that PTEN expression by the virus did not alter the safety profile of the virus in normal cells (Fig. 5d, e). MDA-MB-468 tumor cells were used as controls for the toxicity assay and showed increased sensitivity to HSV-P10 relative to HSVQ.

### HSV-P10 offers a therapeutic benefit and antitumor immunity

**Fig. 6:**
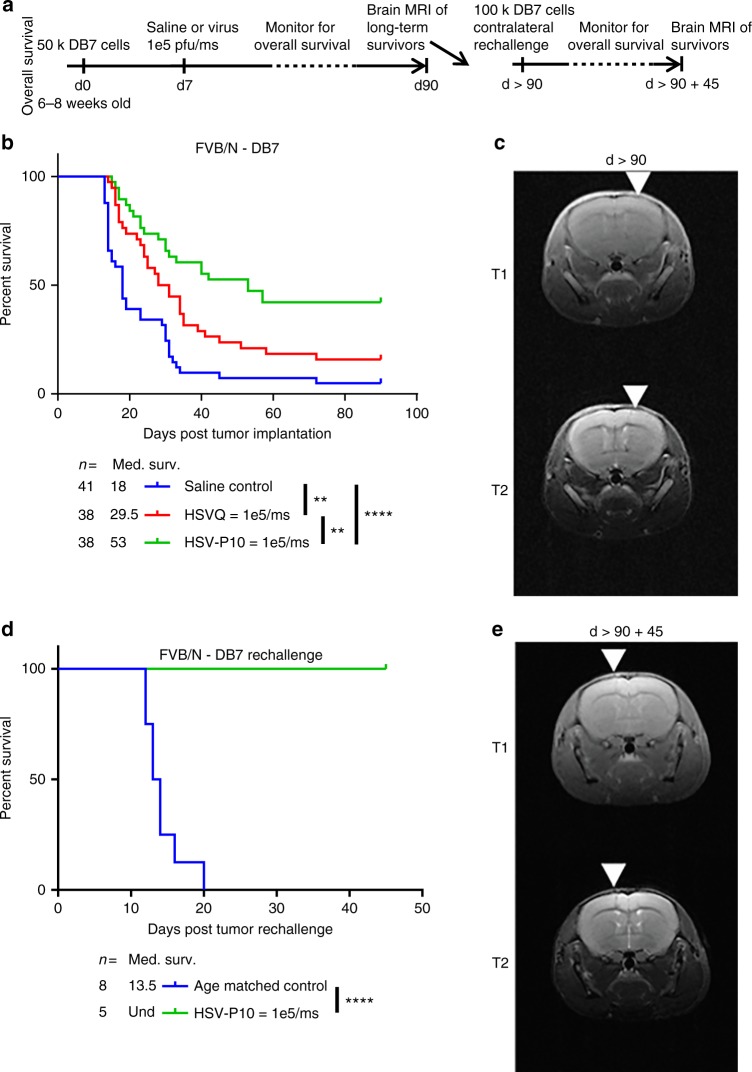
HSV-P10 enhances overall survival of mice bearing breast cancer brain metastases. **a** Schematic representation of animal studies. **b** Survival curve of DB7 brain tumor-bearing FVB/N mice treated intratumorally with saline control (blue line: *n* = 41), HSVQ (red line: *n* = 38), or HSV-P10 (green line: *n* = 38) 7 days post tumor cell implantation. Significance in survival was assessed by Logrank (Mantel–Cox) test comparing only two survival curves per test (**p < 0.01, ****p < 0.0001). **c** Brain magnetic resonance imaging (MRI) of one representative long-term survivor mouse treated with HSV-P10 from Fig. 5b > 90 days post tumor cell implantation. White arrows indicate the needle track of the initial injection site. **d** Survival curve of HSV-P10 treated long-term survivor mice (green line: *n* = 5) from Fig. 5b contralaterally inoculated with 100,000 DB7 tumor cells vs. naive age-matched control mice (blue line: *n* = 8). Significance in survival was assessed by Logrank (Mantel–Cox) test (***p < 0.001). **e** Brain MRI of one representative long-term survivor mouse from Fig. 5d 45 days post tumor rechallenge. White arrows indicate the needle track of the rechallenge injection site

Next, we tested the impact of HSV-P10 on antitumor efficacy, in a murine model of breast cancer brain metastases (Fig. 6b) and intracranial human GBM tumors in nude mice (Supplementary Fig. 3). To evaluate the impact of PTENα on antitumor efficacy, we treated tumor-bearing mice with a single dose of HSVQ or HSVP-10 (Fig. 6a, Supplementary Figure 3a). We observed that while HSVQ treated mice showed an improvement in survival compared to saline control mice, HSV-P10 treatment provided an even greater improvement in overall survival compared to HSVQ treatment (Fig. 6b). Approximately 42% (16 out of 38) of mice treated with HSV-P10 were long-term survivors, showing no indication of tumor burden 90 days post tumor implantation (by MRI). Complete response was confirmed in these mice by MRI imaging (day > 90 after tumor implant), which revealed an absence of visible tumor in the brains of HSV-P10 mice that survived greater than 90 days after tumor implant (Fig. 6c). The presence of long-term survivors after treatment with HSV-P10 in an immune-competent animal model indicated the engagement of the host antitumor immune response in animals treated with HSV-P10. To test the impact of HSV-P10 on antitumor immunity, we assessed the ability of mice treated with HSV-P10 to develop an antitumor immune memory response. MRI confirmed complete responders (survival greater than 90 days) treated with HSV-P10 and tumor-naïve age-matched control mice, were inoculated with 100,000 DB7 cells in the left (contralateral) brain hemisphere. While 100% of the tumor-naive age-matched control mice succumbed to tumor burden, all of the HSV-P10 treated mice rejected the rechallenge and remained tumor free (Fig. 6d). Complete tumor rejection was confirmed by MRI on day 45 following second tumor rechallenge (Fig. 6e).

### HSV-P10 modulates virally induced PD-L1 expression

**Fig. 7:**
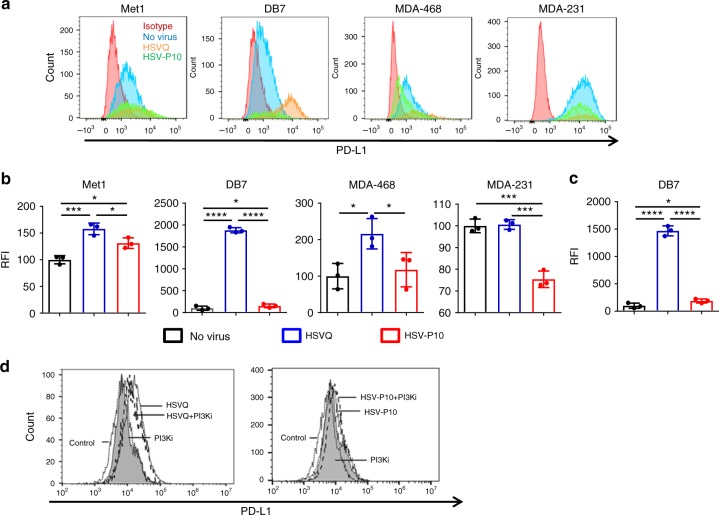
HSV-P10 reduces cell-surface PD-L1 expression. **a** Histograms of PD-L1 expression as measured by flow cytometry 16 hpi in a panel of human and mouse cancer cell lines. Gates were placed on live cells, and then for GFP positive cells. Relative fluoresence intensity was calculated based upon PD-L1 stain. Red histogram: isotype control, blue histogram: no virus, orange histogram: HSVQ, green histogram: HSV-P10. **b** Quantification of relative median fluorescence intensity (RFI) of the indicated cells treated with no virus, HSVQ or HSV-P10 and stained with PDL-1 antibody where statistical significance was assessed by one-way ANOVA (*n* = 3/group, *p < 0.05, ***p < 0.001, ****p < 0.0001). **c** Changes in PD-L1 expression ( ± s.d.) on uninfected (GFP negative) DB7 cells following low-MOI infection with either HSVQ or HSV-P10 where statistical significance was assessed by one-way ANOVA (*n* = 3, *p < 0.05, ****p < 0.0001). Gates were placed on live cells, and then for GFP negative cells. Relative fluoresence intensity was calculated based upon PD-L1 stain. **d** Changes in PD-L1 expression measured by flow cytometry 12 hpi in infected DB7 cells treated with HSVQ ± PI3Ki or HSV-P10 (LY294002). Gating schematics are outlined in supplementary figure 5

PTEN loss has been shown to correlate with PD-L1 upregulation^35,41–43^, and resistance to T-cell mediated therapies^35^. Thus we hypothesized that overexpression of PTENα in tumor cells had the potential to enhance T-cell response to therapy via downregulation of cell-surface PD-L1. To test this, we infected a panel of tumor cell lines with HSVQ or HSV-P10 and measured cell surface PD-L1 expression using flow cytometry (Fig. 7a, b). HSVQ virus infection of tumor cells revealed an induction of cell-surface PD-L1 expression on three of four breast cancer cell lines tested. HSV-P10 treatment showed a reduction of PD-L1 expression relative to HSVQ in all four cell lines tested (Fig. 7a, b). Consistent with the observation of increased PD-L1 observed in melanoma patients receiving oncolytic HSV virotherapy^10^, HSVQ virus infection of tumor cells revealed an induction of cell-surface PD-L1 expression on 3 of 4 breast cancer cell lines tested. HSV-P10 treatment showed a reduction of PD-L1 expression relative to HSVQ in all four cell lines tested (Fig. 7a, b). Analysis of PD-L1 expression in GFP negative uninfected cells revealed a significant induction of PD-L1 expression in cells adjacent to control HSVQ-infected cells. This increased expression of PD-L1 in adjacent uninfected cells was significantly mitigated in cultures treated with HSV-P10 (Fig. 7c). Since PTENα is thought to retain both lipid phosphatase and mitochondria modulating functions we evaluated the contribution of PTENα’s lipid phosphatase function in modulating tumor cell expressed PDL-1. Briefly, DB7 tumor cells were infected with HSVQ or HSV-P10 with or without LY294002, a PI3K antagonist that can reduce pAKT levels, and were evaluated for cell surface PDL-1 expression by fluorescent associated cell cytometry (gating strategy shown in Supplementary Figure 4). Consistent with Fig. 7a, we observed increased PDL-1 expression upon HSVQ infection (Fig. 7d left panel). This induction of cell surface PDL-1 was significantly rescued when cells were treated with HSVQ in combination with the LY294002. PI3K inhibition did not further reduce PDL-1 expression in tumor cells infected with HSV-P10 (Fig. 7d right panel). These results demonstrate that HSV-P10 mediated downregulation of PDL-1 expression is mediated by its ability to modulate PI3K effect.

### HSV-P10 recruits innate and adaptive immune effector cells

**Fig. 8:**
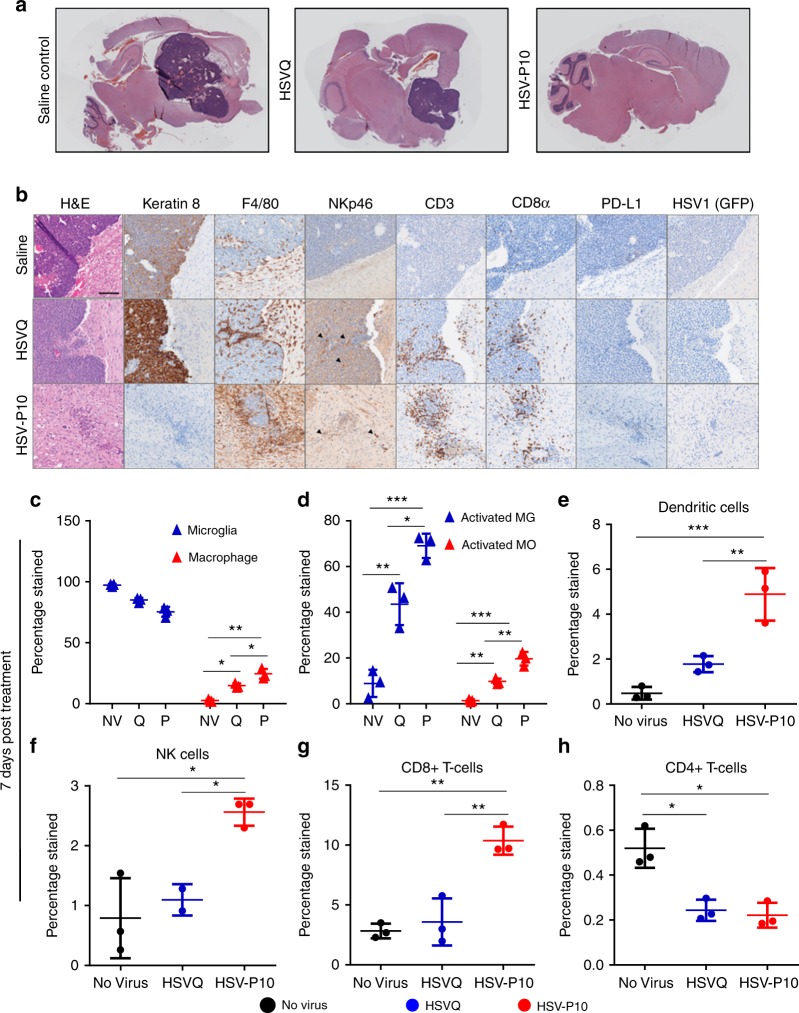
HSV-P10 induces enhanced immune cell influx towards infected tumors. Tumor-bearing FVB/N mice were treated with 1e5 pfu of HSVQ, HSV-P10, or saline control 8 days post tumor implantation. **a** Stitched 4X bright field images of H&E stained sagittal sections of mouse brains 3 days post virus injection. **b** Representative 20X bright field images of H&E, Keratin 8, F4/80, NKp46, CD3, CD8α, PD-L1, and HSV1 (GFP) from sagittal sections of mouse brains 3 days post virus injection. Scale = 0.1 mm. **c** Ratio of macrophages (CD11b + F4/80 + CD45^bright^) to microglia (CD11b + F4/80 + CD45^dim^). **d** Percentage of cells in c positive for MHC-II. **e** Dendritic cell (CD11c + CD80 + MHC-II + ) infiltration. **f** NK cell (CD49b + NKp46 + ) infiltration. **g** CD8 + T-cell (CD3 + CD4 + ) infiltration. **h** CD4 + T-cell (CD3 + CD8 + ) infiltration. Data shown are averages ± s.d (*n* = 3/group) Statistical significance was assessed by one-way ANAOVA (*n* = 3, *p < 0.05, ***p < 0.001, ****p < 0.0001). Gating strategies for (**c**–**h**) are decribed in supplementary figure 6

Because herpesvirus infection, as well as reduced PD-L1, are known to be strong drivers of immunogenic activation^10,17,44–49^, we hypothesized that HSV-P10 infection would have a strong effect on immune cell recruitment and activation in vivo. To evaluate if HSV-P10 altered the immune responses after oncolytic HSV infection, we compared immunohistochemistry (IHC) samples of tumor-bearing mice brains scarified 3 days after virus treatment, which revealed increased immune cell recruitment around virus-treated tumors. H&E staining revealed a gross reduction in tumor burden following HSV-P10 treatment (Fig. 8a). IHC staining for tumor cells expressing cytokeratin revealed the extent of tumor destruction following HSV-P10 treatment (Fig. 8b). Note the absence of cytokeratin 8 (epithelial cell marker) positive tumors in HSV-P10 treated mice 3 days after treatment, highlighting the increased tumor clearance. Both HSVQ and HSV-P10 treated tumors recruited F4/80 + macrophages or microglia. F480 stained cells at these inflamed rims appeared activated with a bushy phenotype which differed from the ramified cells observed at the border between the brain and tumor in the saline treated control group. Both HSVQ and HSV-P10 treatments induced NK cell recruitment (black arrow heads highlight areas of NK cell influx), and staining was observed closely surrounding infected tumors. However, the total number of infiltrating NK cells was much lower in comparison to other immune cell types. CD3 + and CD8 + T-cells were observed preferentially infiltrating the infected tumor mass or tumor remnants, as well as diffusely throughout glial scarring left in the wake of rapid oncolytic tumor lysis. Little to no T-cell staining was observed at distal sites within the brain, indicating that the T-cell response was localized to areas of tumor cell implantation or virus injection. T-cell influx was more pronounced and localized around the microscopic tumor in HSV-P10 treated mice brains, while HSVQ treated tumors had inconsistent areas of high (depicted) T-cell infiltration. We observed a pattern of PD-L1 upregulation in HSVQ treated tumor cells 24 hpi in vitro; however, PD-L1 was not detected in high levels 72 hpi in any of the treatment groups in vivo due to the rapid clearance of virus in this model (Fig. 8b). Both HSVQ and HSV-P10 viruses were observed at detectable levels by both PCR and viral titrations in the brain 24 hpi indicating that PTENα had no antagonist effects on viral replication in vivo (Supplementary Figure 5Figure).

To quantitatively evaluate immune cell recruitment and activation in virally treated animals, we evaluated infiltration of macrophages, NK cells, dendritic cells, and CD4 and CD8 positive T-cells within the tumor-bearing brain hemisphere 7 days post HSVQ or HSV-P10 treatment via flow cytometry (gating strategies depicted in Supplementary Figure 6Figure). Flow cytometric analysis revealed an influx of peripheral immune cells into the brains of virally infected animals compared to saline treated controls (Fig. 8c–h). Treatment with HSV-P10 induced significantly greater macrophage infiltration (CD11b + , F4/80 + , CD45^bright^) (Fig. 8c) and MHC-II presentation on both resident microglia (CD11b + , F4/80 + , and CD45^dim^) (Fig. 8d, blue) and infiltrating macrophages (Fig. 8d, red). HSV-P10 also induced significantly greater dendritic cell (CD11c + , CD80 + , MHC-II + ) (Fig. 8e), NK-cell (CD49b + , NKp46 + ) (Fig. 8f), and CD8 + T-cell (CD3 + , CD8 + ) (Fig. 8g) infiltration over saline control and HSVQ-infected animals. At 7 days post virus infection, ~2.5% of the immune cells in HSV-P10 treated brains were NK cells, and 10% were CD8 + T-cells. Conversely, HSVQ treated animals recruited approximately 1% NK cells and 4% CD8 T-cells, neither of which differed significantly from saline treated control mice. HSVQ infection induced a significant influx in macrophage infiltration (Fig. 8c), and both HSV-P10 and HSVQ treatment resulted in a significant reduction in CD4 + T-cell (CD3 + , CD4 + ) infiltration (Fig. 8h) into infected brain hemispheres. B-cells (CD19 + ) and myeloid derived suppressor cells (CD11b + Gr1 + ) were not detected in any of the treatment groups (data not shown). We therefore assessed the B-cell response via antibody production. To test changes in an antibody-mediated response against virus, tumor, or infected tumor, serum from mice harvested 7 days post virus infection (Supplementary Fig. 7a) was tested for antibody reactivity against purified virus lysate (Supplementary Fig. 7b), DB7 tumor cell lysate (Supplementary Fig. 7c), or HSVQ-infected tumor cell lysate (Supplementary Fig. 7d). While virus infection increased reactivity against tumor, virus, and infected tumor cells, both the viruses induced equivalent anti-virus and antitumor antibody responses.

### Therapeutic benefit of HSV-P10 is T-cell dependent

**Fig. 9:**
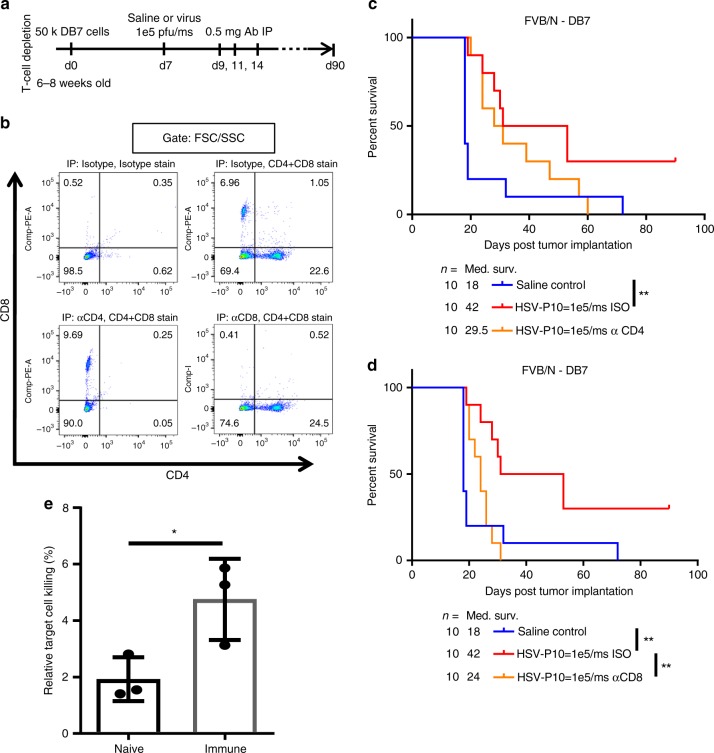
Role for T-cell immunity in virus-mediated tumor clearance. **a** Schematic representation of T-cell depletion study. **b** Confirmation of T-cell depletion by IP anti-CD4, anti-CD8, or isotype treatment 24 h prior to harvesting. Splenocytes were isolated 24 h after IP administration of anti-T-cell depletion antibodies in naïve FVB/N mice, and probed for CD3 + , CD4 + , and CD8 + T-cells as measured by flow cytometry. Live spenocytes were gated using forward and side scatter, and then gated on CD4-FITC and CD8-PE. Single-color positive quadrants (Q1 for CD8 + and Q3 for CD4 + ) were interrogated to determine depletion. **c** Survival of DB7 tumor-bearing FVB/N mice treated with saline control or HSV-P10 7 days post tumor cell implantation, and isotype or anti-CD4 antibodies 2, 4, and 7 days post virus injection. Significance in survival was assessed by Logrank (Mantel–Cox) test (*n* = 10, **p < 0.01). **d** Survival of DB7 tumor-bearing FVB/N mice treated with saline control or HSV-P10 7 days post tumor cell implantation, and isotype or anti-CD8 antibodies 2, 4, and 7 days post virus injection as indicated. Significance in survival was assessed by Logrank (Mantel–Cox) test (*n* = 10, **p < 0.01). **e** ^51^Cr release from uninfected DB7 tumor cells co-cultured with CD3 + splenocytes from untreated naive or immune mice from Fig. 5d. Data shown are averages ± s.d. Statistical significance was assessed by one-tailed Student’s *T*-test (*n* = 3, *p < 0.05)

Considering the observed differences in immune cell recruitment and activation between HSVQ and HSV-P10 treated tumors, we hypothesized that the influx in macrophages and dendritic cells in HSV-P10 treated animals, in addition to the increased MHC-II expression on these cell types, would allow for more robust priming of an antitumor T-cell response. To test our hypothesis that T-cells were primarily responsible for the long-term survival benefit of HSV-P10 treatment, we evaluated the therapeutic benefit of HSV-P10 in mice depleted of CD4 + or CD8 + T-cells using intraperitoneal injections of anti-CD4 or anti-CD8 depleting antibodies (Fig. 9a). Antibody mediated depletion of specific T-cell subtypes was confirmed by flow cytometric analysis of splenocytes of mice treated with either anti-CD4 or anti-CD8 depleting antibodies (Fig. 9b). While CD4 depletion did not affect overall survival of virally treated mice (Fig. 9c, Supplementary Figure 8a), CD8 depletion significantly reduced the survival of HSV-P10 treated animals (Fig. 9d, *p* = 0.0036), but not HSVQ treated (Supplementary Figure 8b, *p* = 0.3109), indicating the importance of CD8 + T-cells in the efficacy of HSV-P10 treatment. T-cell depletion did not affect overall survival of saline or HSVQ treated mice (Supplementary Figure 8c). All mice treated with HSVQ or HSV-P10 with CD8 depletion succumbed to tumor burden, indicating that CD8 + T-cells are essential for the long-term survival benefit of HSV-P10 treatment in vivo. Increased cytotoxicity of T-cells towards tumor cells was further confirmed by a Chromium release assay. T-cells isolated from the spleens of immune DB7 mice showed greater cytotoxicity towards uninfected DB7 cells as measured by ^51^Cr release assay compared to naive control T-cells (Fig. 9e). Combined, these data indicate that mice treated with HSV-P10 are able to generate T-cell-mediated tumor memory response against treated brain tumors.

## Discussion

Oncolytic viruses destroy tumors by infection and cancer cell specific replication and cell lysis. Additionally, the infection induces activation of anticancer immune responses^9,18,19,21,22^. The FDA approval of one such oncolytic herpesvirus, Talimogine laherparepvec (T-VEC), in October 2015 for non-resectable metastatic melanoma has underscored the potential significance of this therapeutic modality. A recent study demonstrated that while T-VEC shows robust immune stimulating capacity in vivo, it also induces the T-cell suppressor molecule PD-L1 on infected cells^10^, which can blunt antitumor immunity post oncolysis^10,28^. Consistent with this observation, recent reports have revealed the potential of combining OV with immune checkpoint inhibitors^10,23–25,28^, implicating that while oncolytic viruses are used as anticancer agents, improvements in virus design could enhance their therapeutic index. Harnessing viruses as vehicles for gene transfer payloads to augment their antitumor efficacy is one way to improve therapeutic index^50^. Here, we sought to boost both the anticancer and immune stimulating properties of an oncolytic virus through the addition of tumor suppressor PTENα.

*PTEN* (and also PTENα) is a commonly lost tumor suppressor gene across a broad range of tumor types^29–31,35,51^. In the brain, metastatic melanoma and breast cancer lesions frequently lose PTEN protein expression, while it is retained in the primary tumor^51^. PTENα is an N-terminally extended form of canonical PTEN, which unlike canonical PTEN, has been shown to be secreted and localize to the mitochondrial membrane^39,40^. As a secreted protein it has the ability to re-enter adjacent cells and retain its function as a lipid phosphatase to reduce PI3K/AKT signaling pathway^39^. HSV1 infection is known to induce AKT phosphorylation during infection through viral proteins VP11 and VP12, suggesting that signaling through the AKT pathway may be important for virus replication^52^. Consistent with this, we also observed increased AKT phosphorylation in tumor cells after infection with control oncolytic HSV. Here we created and tested the efficacy of an oncolytic HSV expressing PTENα. While functionality of PTENα expressed by HSV-P10 as a lipid phosphatase antagonizing PI3K/AKT signaling pathway was confirmed in infected cells, we also observed secretion of PTENα from tumor cells after infection with HSV-P10. Our results showed that HSV-P10 improved the kinetics of virus spread in culture in the cell lines we tested. Since this did not appear to significantly correlate with PTEN status or activated AKT status of the cells in vitro, future studies would focus on uncovering the mechanism by which PTEN expression could augment viral replication.

Interestingly, a recent report suggests that PTENα induces antiviral immunity, resulting in reduced HSV infection and increased clearance by aggravating type 1 IFN responses^53^. In our studies, we did not observe any inhibitory impact of PTENα production by HSV-P10 on virus infection and/or replication. In vivo flow cytometry of brains of mice treated with HSV-P10 treated tumors revealed increased macrophage, dendritic cell, and NK cell infiltration in tumors. PTEN expression correlates with increased NK cell presence, as well as increased macrophage polarization towards an M1 phenotype^17,54–56^. We also observed a significant increase in MHCII positive macrophage recruitment. Taken together, this indicates that while expression of PTEN did not negatively affect virus replication, it did activate innate immunity in vivo, which likely contributed to its therapeutic efficacy.

In the mitochondria, PTENα is thought to induce the activity of mitochondrial cytochrome C, increasing electron transport chain activity and ultimately resulting in the generation of ATP^39,40^. Extracellular ATP released by dead/dying cells is an essential component of immunogenic cell death^40,45–47^, and has been shown to increase antigen presenting cell (APC) recruitment through P2X and P2Y ATP receptors^45,46^. Consistent with these reports, HSV-P10 negatively regulated AKT signaling and increased ATP production and secretion from tumor cells after infection. PTEN loss strongly correlates with PD-L1 overexpression in glioma and colorectal carcinoma^35,41–43,57,58^, thereby driving tumor-reactive T-cells to exhaustion and allowing for immune escape by the tumor^43,59,60^. PTEN expression has also been negatively associated with the development of resistance to anti PD-1 (pembrolizumab) therapy^58^. HSV-P10 infection of tumor cells resulted in loss of infection induced upregulation of PD-L1. Thus PTEN restoration might have the potential to activate antitumor immunity. Here we tested the impact of PTEN gene therapy in conjunction with oncolysis in an immune-competent brain metastases model. A single injection of HSV-P10 led to a statistically significant increase in survival of tumor-bearing mice with almost 50% of the mice displaying a complete response. We tested the ability of HSV-P10 treatment to harness a memory response against tumor cells by re-challenging long-term survivors treated with HSV-P10 with tumor implantation in the contralateral brain hemisphere. All HSV-P10 treated mice rejected the second tumor implantation, while age-matched control mice all succumbed to tumor burden.

**Fig. 10:**
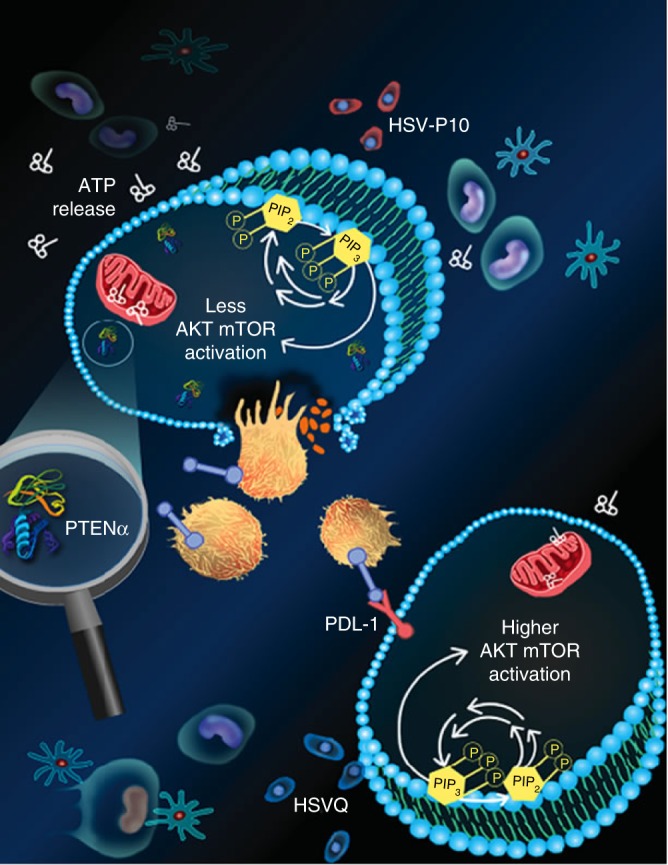
Schematic representation of HSV-P10 antitumor activity: HSVQ (blue) infection (bottom cell) activates AKT/mTOR signaling, initiating the induction of cell surface PDL-1 that provides an immunosuppressive signal to immune cells in the tumor microenvironment. Upon infection with HSV-P10 (red), PTENα expression in infected cells reduces the ratio of Phosphatidyl inositol triphosphate to Phosphatidyl inositol bi phosphate (PIP3/PIP2), and thus consequently negatively regulates AKT/mTOR pathway. PTENα also homes to the mitochondria resulting in increased ATP release from the tumor cells infected with HSV-P10, which activates a robust antitumor immune response. The increased infiltration of activated macrophages, neutrophils, and CD8 + T-cells results in a more efficient tumor cell lysis, and translates to a better therapeutic efficacy

Enhanced therapeutic efficacy and long-term antitumor immunity were accompanied by increased CD8 + T-cell infiltration into HSV-P10 treated tumors, and treatment of mice with the combination of HSV-P10 and anti-CD8 depleting antibody resulted in rescue of the survival advantage (*p* = 0.0036), while the depletion of CD4 + cells in mice did not affect antitumor efficacy of HSV-P10. These data clearly implicate CD8 + T-cells as a major modulator of HSV-P10′s antitumor effects. CD4 + T-cells are responsible for activating B-cells which then mature into plasma cells^61^, and for memory T-cell activation^61^. In both HSVQ- and HSV-P10-treated immunocompetent animals, we detected serum antibodies recognizing both virus and infected and uninfected DB7 tumor cell lysates, indicating that infection with either virus is capable of inducing the maturation of plasma cells. Collectively, our findings demonstrate that HSV-P10 enhances overall survival of brain tumor-bearing mice using a two-pronged approach: lytic tumor cell death accompanied by immune cell education and activation against the tumor. The incorporation of PTENα into an oncolytic virus was able to induce a robust antitumor immune response after a single intratumoral injection, which was shown in vitro to affect tumor cell PD-L1 expression. In conclusion this study demonstrates that HSV-P10 suppresses AKT/mTOR activation, induces ATP release, and decreases PD-L1 cell membrane expression, allowing for an increased CD8 + T-cell mediated antitumor immune response (Fig. 10). Conversely, HSVQ induces AKT/mTOR activity and PD-L1 expression, which diminishes the antitumor immune response observed with HSV-P10 infection (Fig. 10). This observation could pave the way for a paradigm shift in immunotherapy, where immune checkpoint inhibition occurs locally within the tumor rather than globally using neutralizing antibodies. This combination could significantly reduce the cost of therapy and possibly eliminate side effects associated with systemic immunotherapy by requiring only a local injection of the therapeutic virus.

It is important to note that patients with heterozygous PTEN mutations are at risk for developing autoimmunity and lymphoplasia^62^. PTEN expression in Treg cells has been shown to be important for their immunosuppressive phenotype^63^ such that either genetic or pharmacologic ablation of PTEN in this cell population interferes with their suppressive properties. The tumor localized expression of PTEN by HSV-P10 likely bypasses the immunosuppressive effect of PTEN expression in Treg cells, and provides an advantage over its therapeutic modulation of this pathway. Viral infections have the power to divert immune suppressive tumor education of its microenvironment to awaken antitumor immunity: this can be a double-edged sword wherein antiviral effects can also expedite clearance of the viral therapeutic. In general we believe the antiviral effects would be limited to the virus and/or infected cells and could be considered detrimental to virotherapy as they would likely increase virus clearance from the tumor and have minimal effect on uninfected tumor cells. Antitumor immunity would enhance therapeutic benefit by synergizing with the virus for tumor cell clearance. Since we observed a complete rejection of a subsequent uninfected tumor cell challenge in mice that survived HSV-P10 treatment (Fig. 6d), and T-cells isolated from HSV-P10 treated tumor-bearing mice compared to naive mice were able to harness a significantly higher cytotoxicity against uninfected DB7 tumor cells without an enhanced virus clearance in vivo, we believe that antitumor effects contribute to the enhanced antitumor efficacy imparted by HSV-P10.

In conclusion, this study highlights the benefits and translational significance of arming oncolytic herpesviruses with PTENα. Since several GMP facilities including the NIH exist for large-scale development of herepesviruses manufactured to GMP or CE certificate standards, detailed evaluation of safety and biodistribution in two different animal models could be achieved rapidly to translate this therapy for safety and efficacy evaluation in patients.

## Methods

### Virus construction

HSV-P10 was constructed following the HSV-Quik protocol^64,65^. Briefly, a *PTENα* expression cassette with PTENα cDNA driven by the HSV immediate early (IE) promoter IE4/5 was constructed. The cassette was inserted into the disrupted ICP6 locus of an F-strain attenuated HSV bacterial artificial chromosome, driving the expression of PTENα alongside other viral IE genes. BAC DNA was transfected into Vero cells alongside helper plasmids to excise the bacterial origin of replication, and facilitate the production of replication competent virus particles. Clonal expansion was accomplished through plaque purification and amplification in Vero cells.

### Virus purification

A total of 10–25 150 mm dishes were seeded overnight with Vero cells at ~80% confluence in 2% FBS-containing DMEM at 37 °C. Seeded cells were infected with virus (either HSVQ or HSV-P10) at low multiplicity of infection (MOI) and incubated at 34 °C until ready for virus harvest. Cells were pelleted at 500× *g* in a tabletop centrifuge, and cell-containing media was subjected to 3 freeze-thaw cycles (liquid nitrogen, 37 °C water bath) to release virus from cells. Cell debris was pelleted at 4000 RPM and supernatants were combined and transferred to 250 mL centrifuge containers where the virus was pelleted at 13,000 RPM using a floor-standing centrifuge. Virus pellets were resuspended in sterile saline, filtered through a 5 μm Millex PVDF filter, and overlaid on 30% sucrose in sterile saline in Oak Ridge tubes. Virus was re-pelleted at 11,000 RPM using a floor-standing centrifuge, and pellets were resuspended in sterile saline supplemented with 10% glycerol, aliquoted, and stored at −80 °C.

### Virus titration

Virus concentration was measured using a plaque forming assay. Purified virus was serially diluted and added to a 96-well plate. Virus-containing wells were overlaid with 10,000 Vero cells per well and incubated at 37 °C overnight. ≤16 h after Vero cell overlay, pooled human IgG (Grifols) was added at a final concentration of 1 μg/mL, and plates were transferred to 34 °C for 48–72 h to develop plaques. Plaques were counted using fluorescence microscopy, and virus concentration was calculated as plaque forming units (PFU)/mL.

### Cell lines

DB7, Met-1, and MVT-1 murine breast cancer and MDA-MB-231, SK-BR-3, MCF-7, and MDA-MB-468 human breast cancer cells were kindly donated by the lab of Dr. Mike Ostrowski at Ohio State University. U251 cells were obtained from Dr. Erwin G. Van Meir (Emory University, Atlanta, GA), and U251-T3 cells were created in our laboratory (May 2009) as a tumorigenic clone of U251 cells by serially passaging these cells three times in mice. LN229 and U87ΔEGFR cells were obtained from Erwin G. Van Meir (Emory University, Atlanta, GA). U87 cells are listed on the ATCC web site as as “likely glioblastoma cells” of male origin. Human Neural stem cells were obtained from Invitrogen (Carlsbad CA) and were differentiated into neurons per manufacturer protocols. HUVEC were obtained from ScienCell Research Laboratories Carlsbad, CA. U87ΔEGFR, U251-T3, and LN229 human glioblastoma cell lines were verified by short tandem repeat (STR) profiling to match DSMZ-held stocks at all loci on 1/14/2015. Vero cells were purchased from ATCC in 2013. U87ΔEGFR, U251-T3, and LN229 reporter cell lines were generated using lentiviral transduction of either a reporter construct or control construct, followed by puromycin selection for transduced clones. All cells were maintained in Dulbecco’s modified Eagle’s medium (DMEM) supplemented with 10% fetal bovine serum (FBS) and 1% penicillin/streptomycin antibiotic. All cells are routinely STRS profiled, maintained at a passage below 50, and checked for mycoplasma. The UTHSC IRB has determined that these studies qualify for exempt status.

### Animals

All animal experiments were in compliance relevant ethical regulations and were conducted with the approval of the Ohio State University and University of Texas Health Science Center IACUC. For intracranial surgery, mice were anesthetized, heads shaved (if necessary), and stabilized in a stereotactic device (Kopf). The surgical site was sterilized with betadine/isopropyl alcohol, and a midline incision was made in the scalp. A 1 mm burr hole was drilled 2 mm lateral and 1 mm anterior to bregma, and needles (Hamilton 80300 for cells or Hamilton 80000 for virus) were lowered 3 mm into the brain and injected in a 2 µL volume over 5 min using autoinjectors (KD Scientific). Needles were removed, burr holes were filled with bone wax, and scalps were sutured using nylon sutures.

For survival studies, 50,000 DB7 breast cancer or U87ΔEGFR cells were implanted into the brains of 6–8 week old immunocompetent FVB/N (Jackson Labs) or immunocompromised athymic NUDE mice (TVSR, Ohio State University), respectively. Seven days after tumor cell implantation, mice were treated intratumorally with sterile saline control, or 1e5 PFU of either HSVQ or HSV-P10.

For T-cell depletion, mice were injected intraperitoneally with 0.5 mg of isotype control (Bio-X-Cell BE0090), anti-CD4 (Bio-X-Cell BE0119), or anti-CD8a (Bio-X-Cell BE0117) antibodies (Supplementary Data 1) 2, 4, and 7 days following virus injection following previously published protocols^66^. Mice were then monitored for overall survival.

For tumor rechallenge, long-term survivor FVB/N mice (>90 days after tumor implantation) were imaged by MRI for the presence or absence of brain tumor. Mice with no indication of tumor burden were inoculated with 100,000 DB7 tumor cells into the left (contralateral) brain hemisphere alongside age-matched control mice. Mice were then followed for overall survival, and a follow-up MRI was performed 45 days after tumor rechallenge.

For immunohistochemistry, immune cell flow cytometry, and in vivo virus titration, 6–8-week-old FVB/N mice were intracranially inoculated with 100,000 DB7 cells as described. Eight days after tumor cell implantation, tumors were injected with saline control, or 1e5 PFU of either HSVQ or HSV-P10 in a volume of 2 µL. One, three, or seven days after virus injection, mice were euthanized and brains were extracted. For immunohistochemistry, whole brains were fixed with 4% paraformaldehyde and stored at 4 °C for 48 h. For immune cell flow cytometry and in vivo virus titration, tumor-bearing hemispheres were separated from non tumor-bearing hemispheres, and tumor-bearing hemispheres were immediately placed in PBS. Virus titrations were performed on homogenized, freeze/thawed tumor-bearing brain hemispheres using the supernatant. Mononuclear cells were isolated following previously described protocols^67^ for flow cytometric analysis.

For splenocyte isolation, spleens were dissected from euthanized mice and immediately placed in 10% FBS-containing DMEM. Splenocytes were extracted from spleens using the plunger end of a 1 mL syringe, and splenocytes were passed through a 70 µm cell strainer. Strained cells were pelleted, red blood cells lysed in RBC lysis buffer, and re-pelleted to remove lysis buffer and cell debris.

### Western blot

Cultured cells were infected with HSVQ, HSV-P10, or saline at the indicated MOI for the indicated time. Cell lysates were harvested in RIPA cell lysis buffer with protease and phosphatase inhibitors added prior to use. Lysed cells were sonicated and cell debris removed via centrifugation. Protein concentration was measured by BCA and normalized prior to loading in precast gels. PVDF membranes were exposed to antibodies following manufacturer’s protocol. Primary (from CST: PTEN (9559), Akt (4691), pAkt-S473 (9271), from abcam: HSV1 ICP4 (ab6514), GAPDH (ab9484)) and secondary antibodies (from abcam: anti-mouse HRP (ab6789), anti-rabbit HRP (ab6721) can be found in Supplementary Data 1. All phospho-antibody incubations were performed overnight at 4 °C, and all non-phospho-antibody incubations were performed at RT for 1–2 h. Uncropped blots are depicted in Supplementary Figure 9.

### Immunocytochemistry

DB7 or Met1 cells were seeded on glass-bottomed tissue culture dishes overnight. Seeded cells were infected at MOI = 0.1 with either HSVQ or HSV-P10 for 12 h. Infected cells were washed 3×, fixed/permeabilized, and stained for pAkt-S473 using an anti-pAKT primary (CST 4060) and AF-594 tagged secondary (Thermo Fisher A-11012) antibody.

### Cell surface and intracellular flow

For immune cell profiling of tumor-bearing brain hemispheres, mice were euthanized and whole brains were grossly dissected. The tumor-bearing brain hemisphere was dissociated by filtration through a 40 μm cell strainer, and immune cells were isolated in a percoll gradient. Immune cells were then stained for a variety of markers for differentiation (Supplementary Data 1). Macrophages/microglia/myeloid derived suppressor cells were stained using antibodies against CD11b (Miltenyi 130-096-834), F4/80 (Miltneyi 130-102-422), CD45 (Miltenyi 130-102-430), Gr1 (Miltenyi 130-102-385), and MHC-II (BD 563413). B and NK cells were stained with CD19 (Miltenyi 130-102-494), CD49b (Miltenyi 130-102-337) and NKp46 (Miltenyi 130-102-347). T-cells were stained with CD3 (Miltenyi 130-102-793), CD4 (Miltenyi 130-102-541), and CD8 (Mitenyi 130-102-807), and dendritic cells were stained with CD11c (MItenyi 130-102-545), CD80 (BD 560523), CD86 (Miltenyi 130-102-558), and MHC-II BD 563413). Stained cells were analyzed using a flow cytometer (BD LSR-II).

For splenocyte sorting, isolated splenocytes were stained for CD3 (Miltenyi 130-102-793) and NKp46 (Miltenyi 130-102-347) (Supplementary Data 1), and sorted using a flow cytometer (BD FacsAria II).

For intracellular flow cytometry, U87ΔEGFR cells were seeded overnight, and infected at MOI = 0.2 with either HSVQ or HSV-P10 for 8 h. A volume of 5 μM BKM120 (PI3K inhibitor) was used 1 h as a negative control. Treated cells were fixed/permeabilized and stained using an anti-pAKT S473 V450 antibody (BD 560858) (Supplementary Data 1). Stained cells were analyzed using a flow cytometer (BD LSR-II).

FACS sequential gating/sorting strategies are shown for Fig. 5 d,e in supplementary figure 2, for Fig. 7d in supplementary figure 5, and for Fig. 8c-h in supplementary figure 6. FACS gating strategies are described verbally in the figure legends of Fig. 7 a,b and of Fig. 9b.

### Viral infection and replication assay

Virus titration was performed on Vero cells using a plaque forming assay. In vitro, tumor cells were seeded overnight and infected at a MOI of 0.005. Cells and media were harvested 72 hpi. Cells were lysed either by sonication or freeze/thaw cycles, and cell debris was pelleted and removed. Serial dilutions of virus-containing media were overlaid with Vero cells and incubated at 37 °C for ≤ 16 h. Pooled human immunoglobulin was added at 1 μg/mL final concentration, and cells were transferred to 34 °C for 48–72 h to develop plaques. In vivo, FVB/N mice were intracranially inoculated with 100,000 DB7 cells as described. Eight days after tumor cell implantation, tumors were injected with a single dose of 1e5 PFU of either HSVQ or HSV-P10. Mice were euthanized, and tumor-bearing brain hemispheres were collected, homogenized via filtration through a 70 µm cell strainer, and sonicated for viral release. Cell debris was removed via centrifugation and supernatant was serially diluted and assayed as described above.

To measure infection and virus replication over time, cells were seeded at a density of 5 × 10^4^ cells per well in 24-well plates overnight. Seeded cells were infected with 0.05 MOI of HSVQ or HSV-P10, and virus infection was quantified by GFP expression, which was monitored utilizing the Cytation 5 Cell Imaging Multi-Mode Reader (Biotek) where images were taken every 2 h.

### Cell proliferation assay

Cells were seeded in 96-well plates containing 5000 cells/well in 2% FBS-containing media. Seeded cells were infected at MOI = 0.5 with either HSVQ or HSV-P10 in 2% FBS-containing media. Cells and virus were incubated for 24–96 hpi at 37 °C, and cell viability was measured using a 2-step MTT assay (Roche) following manufacturer’s protocol. Absorbance was measured using a spectrophotometer (BMG Labtech FLUOstar Optima), and cell viability was measured as the percentage of uninfected control.

### ATP measurement

Tumor cells were seeded in 24-well plates containing 100,000 cells per well in 2% FBS-containing media. Cells were allowed to adhere to tissue culture plates overnight at 37 C, and were infected at MOI = 0.5 with HSVQ or HSV-P10 for 1 h. Unbound virus was removed and media was replaced. 24 hpi, conditioned media was harvested and ATP concentration was assessed using an ATP determination kit (Thermo Fisher). Cell lysates were collected in RIPA buffer with protease and phosphatase inhibitors, and protein concentration was determined by BCA assay (Pierce, Thermo Fisher). Equal amounts of protein were then analyzed for ATP concentration as described. For OV ± AKTi measurements, cells were seeded in 24-well plates at a density of 200,000 cells/well in 2% FBS-containing media, and infected at MOI = 0.1 with HSVQ or HSV-P10 for 1 h. Unbound virus was removed and replaced with media ± 5 µM LY294002 (a PI3K inhibitor that reduces AKT phosphorylation). Conditioned media was harvested 12 hpi and ATP was measured via CellTiter-Glo 2.0 (Promega).

### ^51^Cr release assay

Splenocytes from naive or immune animals were harvested and sorted as described. CD3 + cells were stimulated by CD3/CD28 beads (Gibco) and recombinant IL2, and co-cultured with ^51^Cr labeled uninfected DB7 cells. Maximum (SDS treatment) and minimum (no co-culture) ^51^Cr release controls were included for analysis, and cells were incubated at 37 C overnight. Cells were then collected via centrifugation and supernatants were measured for ^51^Cr concentration using a scintillation counter.

### ELISA

For antibody titrations against virus, infected DB7 cells, and uninfected DB7 cells, mouse serum was harvested from tumor-bearing FVB/N mice 7 days post virus treatment by submandibular bleed, followed by coagulation and separation of whole blood, and collection of serum. Opaque 96-well plates were coated with antigen in carbonate/bicarbonate buffer overnight at 4 °C, and washed 4 times with PBST. Serum was diluted in 5% skim milk/PBST 1:100, incubated 2 h at RT. Plates were washed 4 times with PBST, and incubated with anti-mouse HRP at 1:1000 in 5% skim milk/PBST for 1 h at RT. Plates were washed 4 times with PBST, and substrate added for quantification using a spectrophotometer (BMG Labtech FLUOstar Optima).

### Immunohistochemistry

Dissected tissues were fixed in 4% paraformaldehyde solution for 48 h, dehydrated in 30% sucrose for 24 h, and transferred to 70% ethanol. Tissues were processed, embedded in paraffin, cut in 5 μm sections on positively charged slides, de-paraffinized, rehydrated, and stained with H&E.

For immunohistochemistry, all sections were stained using a Bond Rx autostainer. Briefly, slides were heated at 65 °C for 15 min and the automated system performed dewaxing, rehydration, antigen retrieval, blocking, primary antibody (Cytokeratin 8 (Developmental Studies Hybridoma Bank, University of Iowa), CD31 (Santa Cruz sc-1506R), PTEN (CST 9559), F4/80 (Invitrogen MF48000), NKp46 (Abcam ab214468), CD3 (Abcam ab16669), CD8a (CST 98941), PD-L1 (CST 64988)) incubation, post primary antibody incubation, detectionco, and counterstaining using Bond reagents. Samples were then removed from the machine, dehydrated with ethanols and xylenes, mounted and coverslipped. Antibodies were diluted in antibody diluent, as indicated in Supplementary Data 1.

### Magnetic resonance imaging

Micro MRI imaging was conducted using a 9.4 T BioSpec 94/30 Imaging System. T1 and T2 weighted images were captured and analyzed using Paravision 5.1 software. Screen captures of coronal brain sections at the site of tumor injection (as seen by needle track) were included for publication.

### Statistical calculations

For ATP quantification and comparison (Fig. 3b, c, e) and ^51^Cr release assays (Fig. 9e) 2-tailed Student’s *T*-test at *p* = 0.05 were performed. Assays requiring multiple comparisons including viral infection and PD-L1 quantification (Fig. 7b, c), immune cell tumor infiltration quantification (Fig. 8 c-h), and antiviral antibody titer quantifications (Fig. S5 b-d) were analyzed by one-way ANOVA. All survival studies were analyzed using a Logrank (Mantel–Cox) test comparing only two survival curves at a time.

## Electronic supplementary material

Supplementary Information

Description of Additional Supplementary Files

Supplementary Data 1

## Data Availability

The data that support the findings of this study are available from the corresponding author upon reasonable request.
