# Supplementary material for: PTEN expression by an oncolytic herpesvirus directs T-cell mediated tumor clearance
Source: Nat Commun. 2018 Nov 27;9:5006. doi: 10.1038/s41467-018-07344-1 (PMC6258708; doi:10.1038/s41467-018-07344-1)
Supplement: Supplementary file 1 — Supplementary Information [file 41467_2018_7344_MOESM1_ESM.pdf]

PTEN expression by an oncolytic herpesvirus directs T-cell mediated tumor clearance

Russell et al.

Supplementary Figure 1

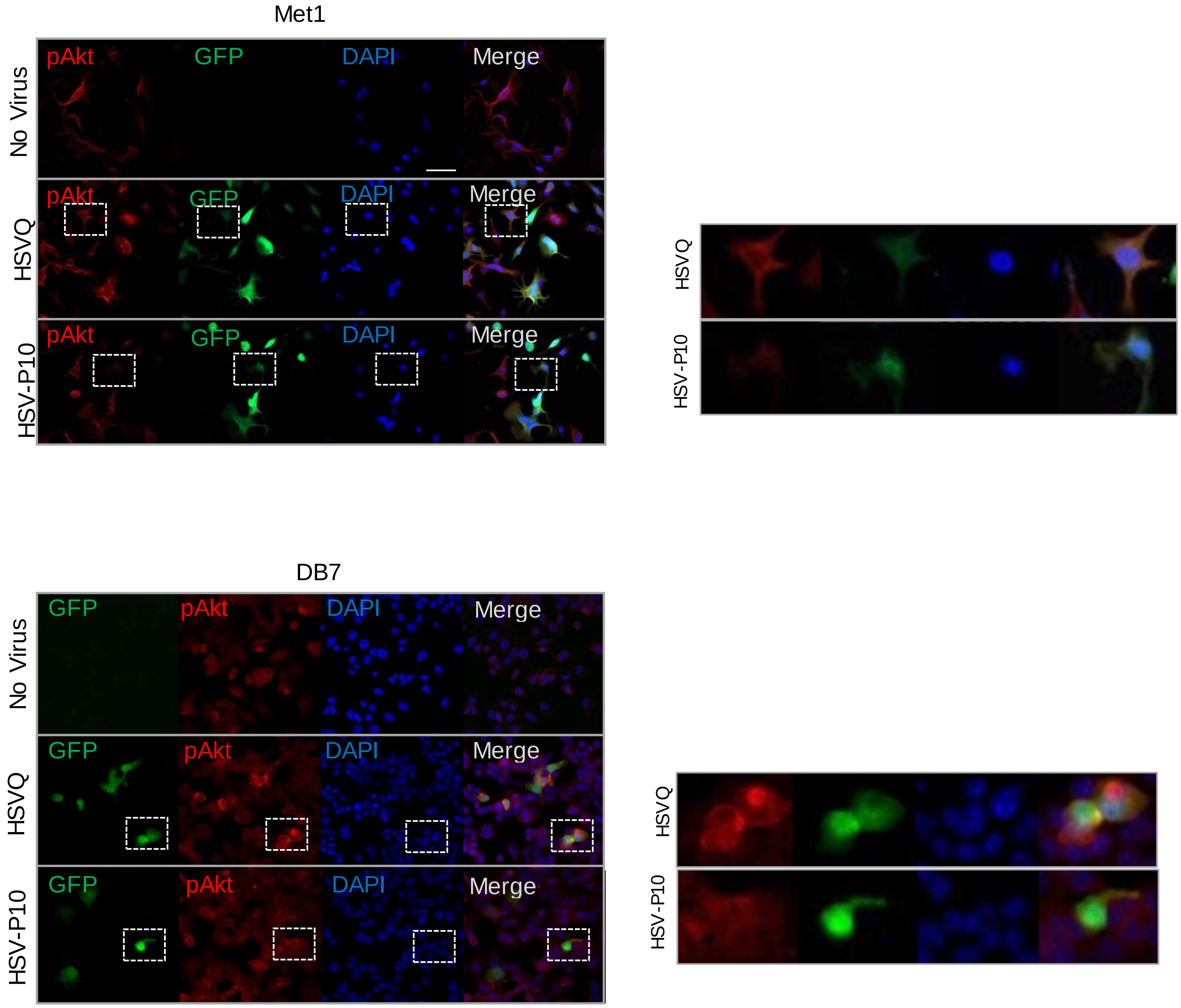

Supplementary Figure 1: AKT activity in infected Met1 and DB7 cells. Immunocytofluorescent confocal microscopy of pAkt (red), viral GFP (green), and DAPI (blue) in Met1 and DB7 cells 12hpi. Panel on right shows magnification of inset area in left panel. Scale bar is 100µm.

Supplementary Figure 2

a

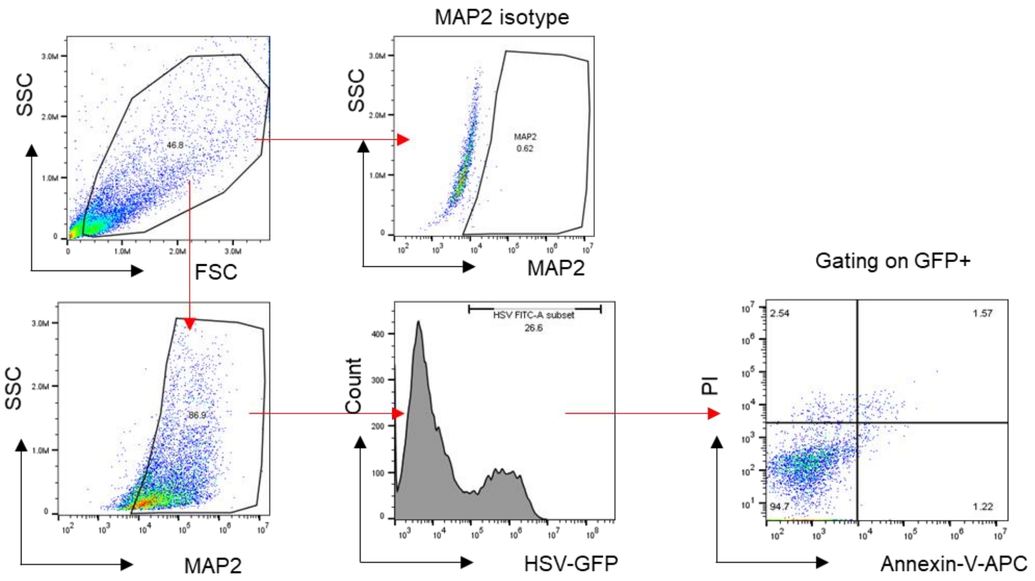

b

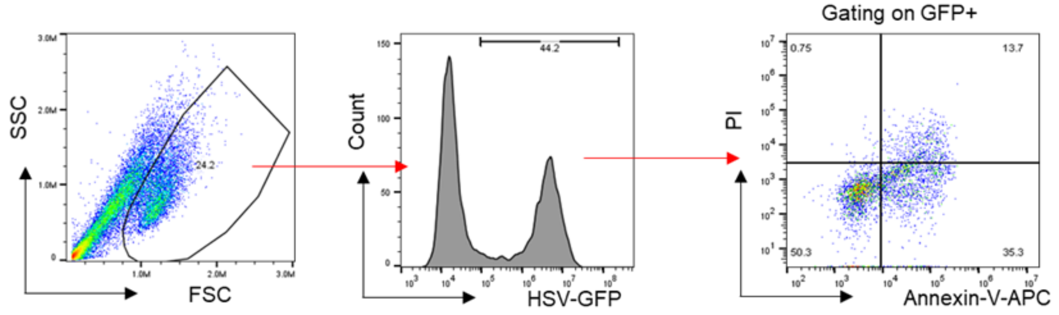

Supplementary Figure 2: Flow cytometry gating strategies-Figure 5d-e. Neurons and MDA-468 tumor cells were infected with HSVQ or HSV-P10 and analyzed for infection (HSV-GFP) and cell death (PI and Annexin-V-APC). Gating schematics for a) neurons and b) MDA-468 tumor cells are depicted for HSV infection and cell death.

Supplementary Figure 3

a

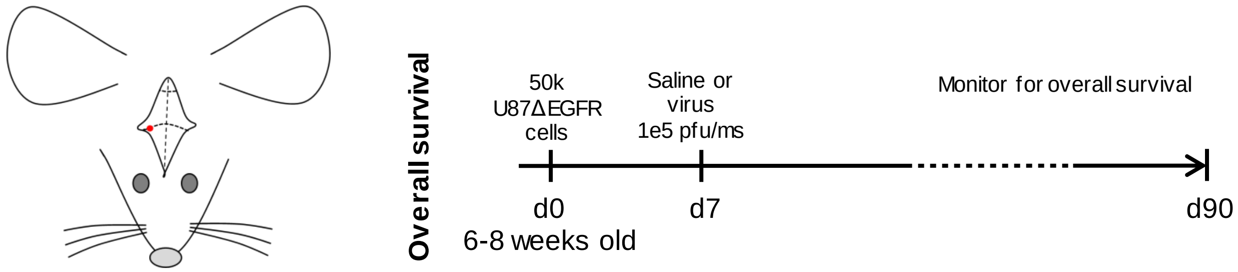

b

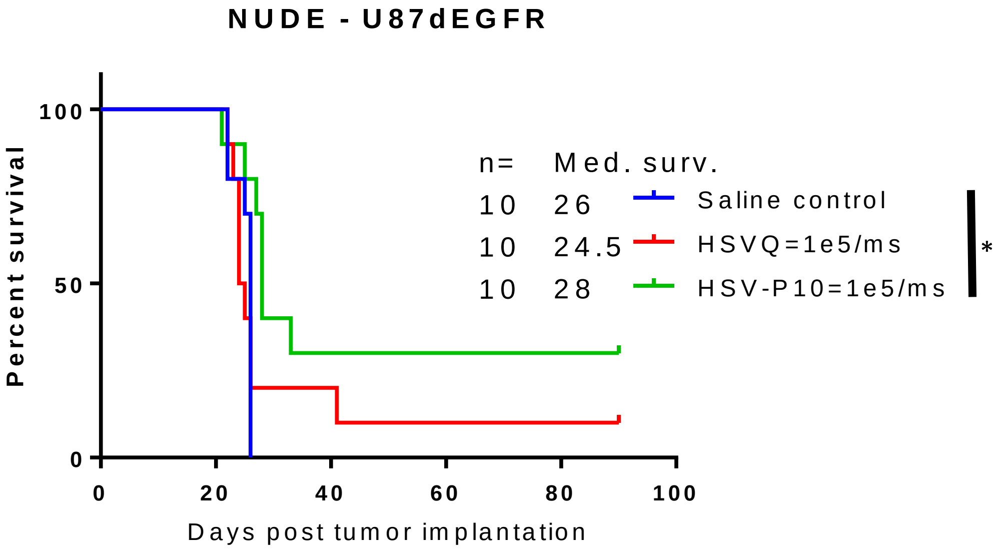

Supplementary Figure 3: Survival of immunodeficient mice bearing intracranial U87ΔEGFR treated with HSVQ or HSV-P10. a) Schematic representation of time line of tumor implantation and treatment of mice. Mice were treated with the indicated virus on day 7 and with (10/group). b) Overall Kaplan Meier survival curves of mice bearing intracranial U87ΔEGFR tumors treated as indicated where statistical significance was assessed by Gehan-Breslow-Wilcoxon test (n=10/group, *$p<0.05$). Blue: saline, red: HSVQ, green: HSV-P10.

Supplementary Figure 4

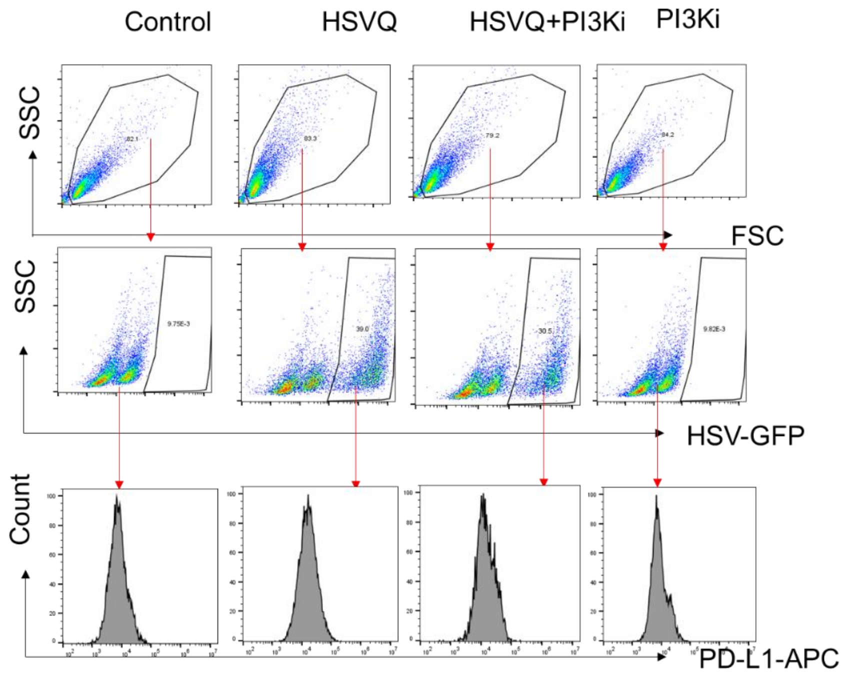

Supplementary Figure 4: Flow cytometry gating strategies-Figure 7d. Flow cytometry of DB7 cells treated with HSVQ, HSV-P10, PI3Ki, or a combination of OV and PI3Ki. PD-L1 expression was assessed by flow cytometry 12 hpi. Gating strategies are shown for Figure 7d, where HSVQ and HSV-P10 histograms were gated identically.

Supplementary Figure 5

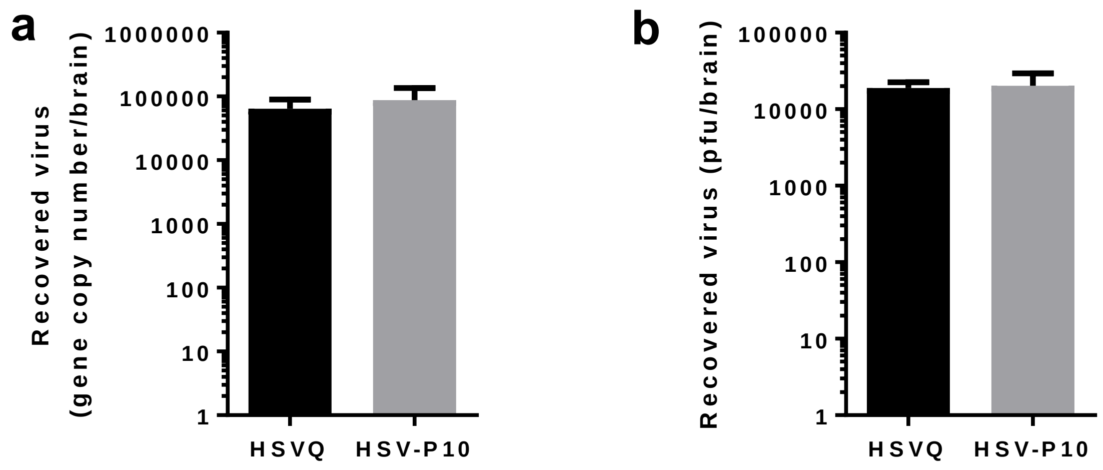

Supplementary Figure 5: HSVQ and HSV-P10 in vivo virus recovery. Tumor-bearing FVB/N mice were treated with 1e5 pfu of HSVQ or HSV-P10 8 days post tumor implantation. Mice brains were harvested 24h post treatment and virus recovery was quantified via a) qPCR of HSV1-gD (n=3) and b) viral titers (n=6) obtained via plaque forming assays.

Supplementary Figure 6

a

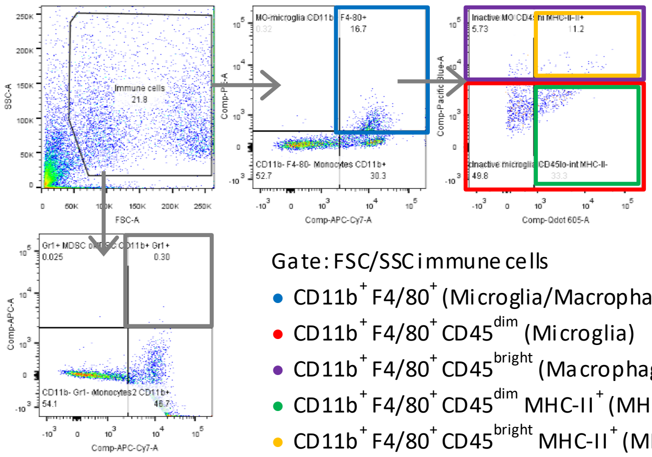

Gate: FSC/SSC immune cells

- CD11b$^+$ F4/80$^+$ (Microglia/Macrophages)
- CD11b$^+$ F4/80$^+$ CD45^{dim} (Microglia)
- CD11b$^+$ F4/80$^+$ CD45^{bright} (Macrophages)
- CD11b$^+$ F4/80$^+$ CD45^{dim} MHC-II$^+$ (MHC-II+ microglia)
- CD11b$^+$ F4/80$^+$ CD45^{bright} MHC-II$^+$ (MHC-II+ macrophages)
- CD11b$^+$ Gr1$^+$ (Myeloid derived suppressor cells)

b

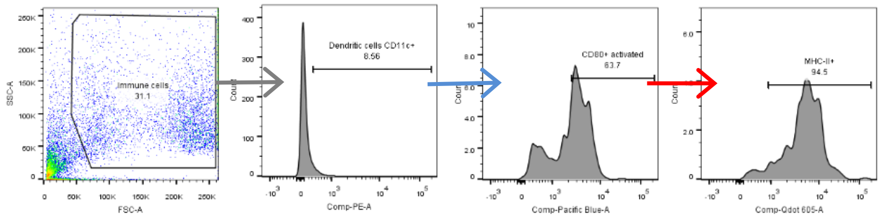

Gate: FSC/SSC immune cells

- CD11c$^+$
- CD11c$^+$ CD80$^+$
- CD11c$^+$ CD80$^+$ MHC-II$^+$ (CD80+ Dendritic cells)

c

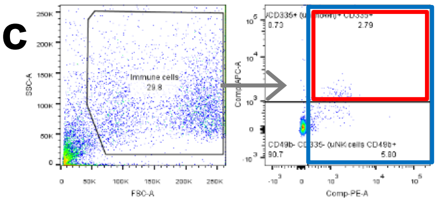

Gate: FSC/SSC immune cells

- CD49b$^+$
- CD49b$^+$ NKp46$^+$ (NK cells)

d

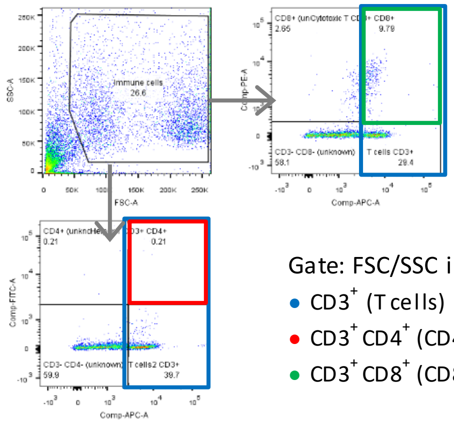

Gate: FSC/SSC immune cells

- CD3$^+$ (T cells)
- CD3$^+$ CD4$^+$ (CD4+ T cells)
- CD3$^+$ CD8$^+$ (CD8+ T cells)

Supplementary Figure 6: Flow cytometry gating strategies-Figure 8c-h. Tumor-bearing FVB/N mice were treated with 1e5 pfu of HSVQ, HSV-P10, or saline control 8 days post tumor implantation. Mice brains were removed 7 days post treatment and immune cells were isolated and stained for flow cytometry. Strategies depicted are for data shown in Figure 8c-h. a) Gating strategy for macrophages, microglia, and myeloid derived suppressor cells. b) Gating strategy for dendritic cells. c) Gating strategy for NK cells. d) Gating strategy for T-cells.

Supplementary Figure 7

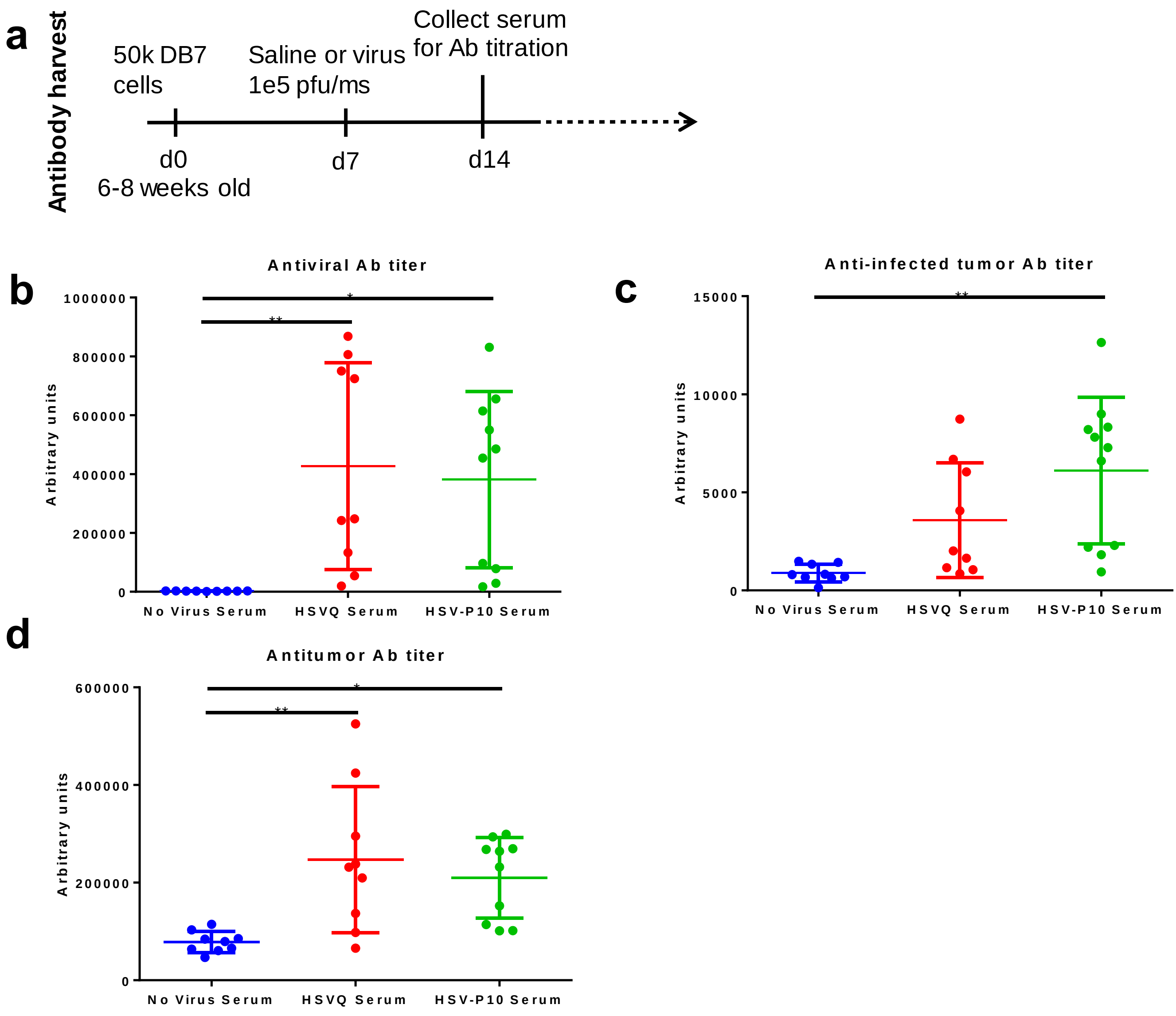

Supplementary Figure 7: Antibody response to virus therapy. a) Schematic representation of animal study. b) Relative serum antibody titers against purified HSVQ lysate using serum harvested from FVB/N mice treated as indicated. c) Relative antibody titer against HSVQ-infected DB7 cell lysate using serum harvested from FVB/N mice treated as indicated. d) Relative antibody titer against DB7 cell lysate using serum harvested from FVB/N mice treated as indicated. Blue: saline, red: HSVQ, blue: HSV-P10.

Supplementary Figure 8

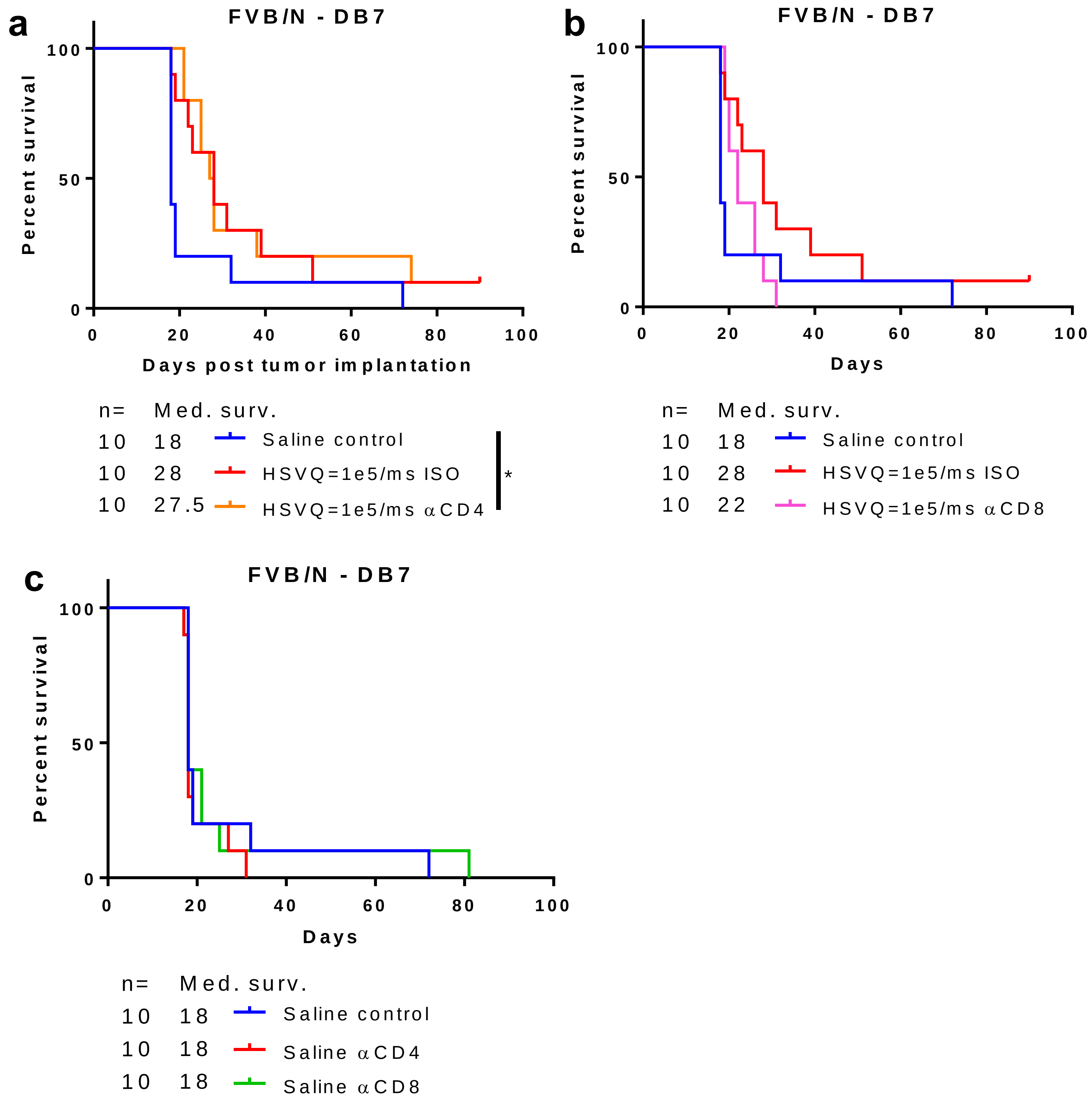

Supplementary Figure 8: Effect of T-cell depletion on HSVQ and saline treatment of tumor-bearing FVB/N mice. a) Effect of CD4+ T-cell depletion on HSVQ treated DB7 tumor-bearing FVB/N mice (n=10/group). Blue: saline, red: HSVQ + isotype control, orange: HSVQ + αCD4. b) Effect of CD8+ T-cell depletion on HSVQ treated DB7 tumor-bearing FVB/N mice (n=10/group). Blue: saline, red: HSVQ + isotype control, pink: HSVQ + αCD8. c) Effect of T-cell depletion on saline treated DB7 tumor-bearing FVB/N mice (n=10/group). Statistical significance was assessed by Logrank (Mantel-Cox) test. Blue: saline, red: saline αCD4, blue: saline αCD8

Supplementary Figure 9

a

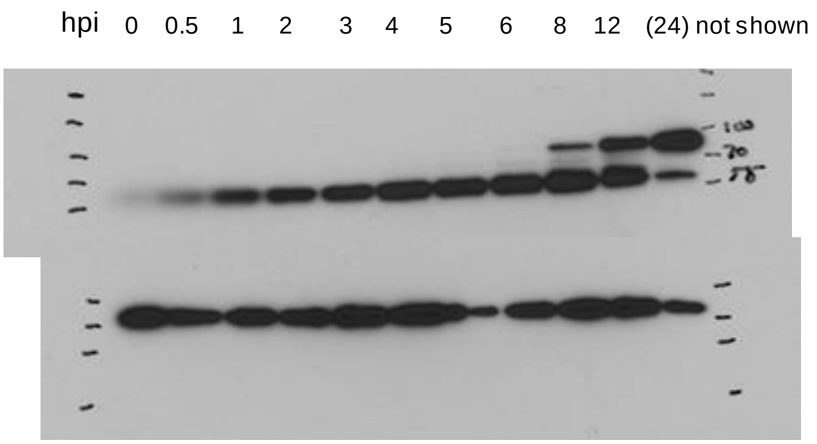

b

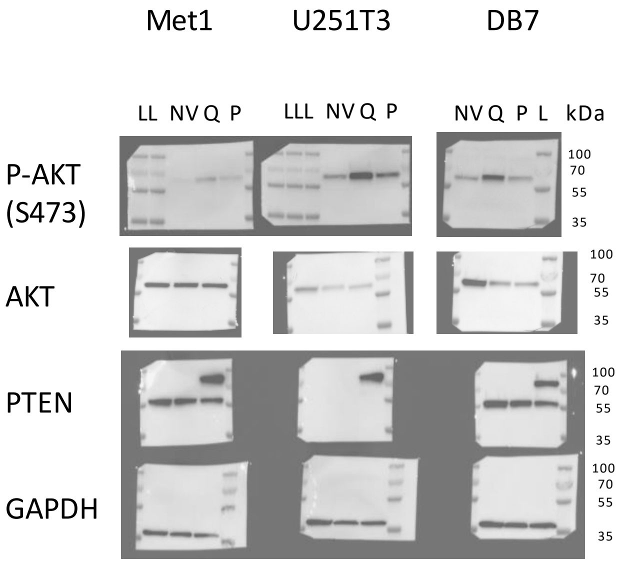

Supplementary Figure 9: Uncropped western blots. a) DB7 cells treated with 0.5 MOI HSV-P10 and PTENα expression was determined over time. b) Met1, U251T3, and DB7 cells treated with 1 MOI of HSVQ (Q) or HSV-P10 (P10) or left untreated (NV), where P-AKT (S473), AKT, PTEN, and GAPDH expression were determined. Molecular weight ladders (L) are shown on all blots.

Supplementary Table 1: PTEN status of cell lines

Tissue of Origin	Cell Line	PTEN Status	PMID
Breast (human)	MDA-MB-468	Mutant	17314276
Breast (human)	MDA-MB-231	Wild Type	17088437
Breast (murine)	DB7	Wild Type, overexpressing PyMT	16132578
Breast (murine)	MET-1	Wild Type, overexpressing PyMT	16132578
Breast (murine)	MVT-1	Wild Type	28430642
Endothelial Cells (human)	HUVEC	Wild Type	11784722
GBM (human)	U251-T3	Mutant	20113523
GBM (human)	U87ΔEGFR	Null	28094268
GBM (human)	LN229	Wild Type	20113523
GBM (murine)	GL261	Mutant	28094268
