# Supplementary material for: PTEN expression by an oncolytic herpesvirus directs T-cell mediated tumor clearance
Source: Nat Commun. 2018 Nov 27;9:5006. doi: 10.1038/s41467-018-07344-1 (PMC6258708; doi:10.1038/s41467-018-07344-1)
Supplement: Supplementary file 2 — Description of Additional Supplementary Files [file 41467_2018_7344_MOESM2_ESM.pdf]

Description of Additional Supplementary Files

File Name: Supplementary Data 1

Description: Antibodies use for western blots (grey), immunocytochemistry (ICC, green), immunohistochemistry (IHC, orange), flow cytometry (blue), and T-cell depletion (pink) studies are specified, along with the species reactivity, isotype used, manufacturer, catalog number, and dilution utilized.
